# Permeation of a Homologous Series of NBD-Labeled Fatty Amines through Lipid Bilayers: A Molecular Dynamics Study

**DOI:** 10.3390/membranes13060551

**Published:** 2023-05-25

**Authors:** Hugo A. L. Filipe, Luís M. S. Loura, Maria João Moreno

**Affiliations:** 1 Coimbra Chemistry Center, Institute of Molecular Sciences (CQC-IMS), University of Coimbra, 3004-535 Coimbra, Portugal; lloura@ff.uc.pt (L.M.S.L.); mmoreno@ci.uc.pt (M.J.M.); 2 CPIRN-IPG—Center of Potential and Innovation of Natural Resources, Polytechnic Institute of Guarda, 6300-559 Guarda, Portugal; 3 CNC—Center for Neuroscience and Cell Biology, University of Coimbra, 3004-535 Coimbra, Portugal; 4 Faculty of Pharmacy, University of Coimbra, 3000-548 Coimbra, Portugal; 5 Department of Chemistry, Faculty of Sciences and Technology, University of Coimbra, 3004-535 Coimbra, Portugal

**Keywords:** amphiphilic molecules, asymmetric lipid bilayers, lipid membranes, molecular dynamics simulations, nitrobenzoxadiazole, permeation, potential of mean force, umbrella sampling

## Abstract

Permeation through biomembranes is ubiquitous for drugs to reach their active sites. Asymmetry of the cell plasma membrane (PM) has been described as having an important role in this process. Here we describe the interaction of a homologous series of 7-nitrobenz-2-oxa-1,3-diazol-4-yl (NBD)-labeled amphiphiles (NBD-Cn, *n* = 4 to 16) with lipid bilayers of different compositions (1-palmitoyl, 2-oleoyl-*sn*-glycero-3-phosphocholine (POPC):cholesterol (1:1) and palmitoylated sphingomyelin (SpM):cholesterol (6:4)), including an asymmetric bilayer. Both unrestrained and umbrella sampling (US) simulations (at varying distances to the bilayer center) were carried out. The free energy profile of NBD-Cn at different depths in the membrane was obtained from the US simulations. The behavior of the amphiphiles during the permeation process was described regarding their orientation, chain elongation, and H-bonding to lipid and water molecules. Permeability coefficients were also calculated for the different amphiphiles of the series, using the inhomogeneous solubility-diffusion model (ISDM). Quantitative agreement with values obtained from kinetic modeling of the permeation process could not be obtained. However, for the longer, and more hydrophobic amphiphiles, the variation trend along the homologous series was qualitatively better matched by the ISDM when the equilibrium location of each amphiphile was taken as reference (Δ*G* = 0), compared to the usual choice of bulk water.

## 1. Introduction

On their way from the administration site to the target active site, drugs need to permeate through biomembranes. Passive transport of drugs through membranes is generally considered the main process of permeation, limiting their penetration into cells and thus being a key step in their availability at a target site, although the requirement of carrier-mediated transport is also claimed [1,2,3]. The inability of molecules to cross tight endothelia such as the blood-brain barrier (BBB) is a major drawback for the discovery of new drugs to treat Central Nervous Systems (CNS) disorders, with important social and economic implications. Following this subject, the kinetic and thermodynamic characterization of the interaction of amphiphiles with lipid bilayers is important to predict the interaction of amphiphilic drugs with biological membranes, a property that determines their pharmacokinetics and bioavailability.

Our team has characterized experimentally the kinetics of the interaction of several amphiphiles with bilayers of different lipid compositions [4,5,6,7,8,9,10]. From the parameters obtained, it is possible to calculate the rate of passive permeation through biological membranes [11]. Overton’s rule [12], which is at the basis of most models for the calculation of passive permeation across biomembranes, assumes the diffusion through the lipid bilayer as the rate limiting step and predicts a positive linear dependence of the permeability coefficient through a cell monolayer with the partition coefficient. For amphiphilic drugs, this diffusion step corresponds to their translocation across the bilayer. Therefore, the determination of the translocation rate constant and the partition coefficient of a drug into a lipid bilayer may be used to determine its rate of passive permeation. This approach has been used by our group for the case of the drug chlorpromazine, with good agreement with the permeability coefficients obtained in vitro with cell monolayers [8]. The characterization of different (but structurally related) amphiphiles may lead to the establishment of rules to predict the rate of passive permeation from the structure of the amphiphile.

In addition to permeability, the equilibrium position of specific drugs in target membranes also affects their metabolism. The free-energy profiles of a set of 25 drug-like molecules in a 1,2-dioleoyl-snglycero-3-phosphocholine (DOPC) bilayer showed that the molecules accumulate in the membrane environment, with the majority lying just below the polar head group region [13]. The localization of drugs on lipid bilayers might affect their interaction with drug-metabolizing cytochrome P450 (CYP) enzymes [14], and as a consequence affect the metabolism of drugs. Further, the localization and affinity to a membrane may play an important role in other biologically significant processes, such as interaction with other membrane proteins [15,16,17,18], and antioxidant inhibition of lipid peroxidation [19,20,21].

The importance of drug-membrane interactions in biology, pharmacology, and medicine has called for extensive research, which is rather challenging due to the complexity of biological membranes. Biological membranes have complex mixtures of lipids and proteins in approximately equal mass proportions [22,23], the former being determinant for the rate of passive permeation. The membrane that has a stronger impact on drug bioavailability is the cell plasma membrane, through which drugs must penetrate to reach the internal milieu of target cells and tissues. The most abundant lipids in mammalian membranes are phosphatidylcholines (PC), although phosphatidylserines (PS), phophatidylethanolamine (PE), sphingomyelins (SM), and cholesterol (Chol) are also present in relatively high amounts. Composition asymmetry across the two bilayer leaflets is a striking feature in eukaryotic plasma membranes [24]. Sphingolipids are almost exclusively distributed in the exoplasmic leaflet, whereas the cytoplasmic leaflet contains large amounts of glycerophospholipids [25]. Cholesterol equilibration between both membrane leaflets is a relatively fast process [26,27,28,29], and therefore, its distribution between both leaflets depends on their phospholipid composition. Due to the higher affinity of cholesterol for SM, and its lower affinity for PE and phospholipids with polyunsaturated acyl chains [6,30,31], a higher concentration of cholesterol is expected in the outer leaflet of the plasma membrane. For instance, in the erythrocyte membrane [24,32,33,34,35,36,37], most SM is located in the outer leaflet [24,33,34], while cholesterol is distributed in both leaflets [24,38,39]. The acyl chains of the inner leaflet lipids have higher unsaturation levels [23], this monolayer being also in a more fluid state [34]. This bilayer asymmetry should be included in the models used to understand and predict the rate of passive permeation of amphiphiles through biological membranes. The use of intact biological membranes may not be a good alternative to obtain the rules of passive permeation due to their intrinsic complexity that may generate case-specific exceptions only valid for each particular membrane and drug. Although important advances have been achieved in the last years [40,41], the experimental preparation of stable asymmetric membranes is very difficult, which makes theoretical work very important in this kind of system.

Molecular Dynamics (MD) is a powerful tool to study the interaction of amphiphiles with lipid bilayers as it gives atomistic details that often cannot be obtained experimentally. Additionally, MD simulations may be performed on asymmetric bilayers. In fact, MD has proved successful in the simulation of stable asymmetric bilayers [42,43,44,45,46]. For membrane permeability studies, MD simulations using the Umbrella Sampling (US) [47,48] technique have been applied to a long list of solutes of increasing size and complexity [49,50,51,52]. The most common application of US to studies of solute interaction in a lipid bilayer uses the displacement of the solute along the bilayer normal as the reaction coordinate to compute the potential of mean force (PMF) for the insertion of the solute from bulk water to the bilayer center [53]. Such studies can be used to predict the conformations associated with the lowest-energy insertion depths, as well as on-pathway intermediates or transition states for solute insertion/desorption or translocation.

The penetration properties of drug-like molecules on lipid bilayers can be well described by considering the free energy profile along the bilayer normal, also called the PMF. The PMF of solutes through lipid bilayers measures the free energy cost (Δ*G*) of moving the solute over a specific distance away from its equilibrium position in the membrane. The free energy minimum on this profile shows the energetically most favorable position of the molecule on the bilayer, which mainly depends on its structural molecular properties. Polar molecules have their most probable location at an energy minimum at the membrane/water interface, while nonpolar molecules prefer to be closer to the bilayer center. For polar molecules, as the molecule proceeds further into the lipid bilayer, the hydrophobicity of the membrane environment increases and the free energy rises, and thus the molecule must overcome an energy barrier. The rate of transfer of the molecules between lipid leaflets is related to the energy barrier at the bilayer center, while the membrane/water barrier reflects the affinity to the bilayer in comparison to the water environment. In the bilayer center, a local energy minimum is sometimes also observed [29,54], and the molecules can reside here for some time [55]. In the aqueous environment, on both sides of a membrane, the free energy of a molecule is constant when the molecule is far enough from the bilayer. For symmetric lipid bilayers, the free energy profile is usually calculated for one leaflet, and the other leaflet is plotted symmetrically. However, for an asymmetric bilayer, sampling of the reaction path must be done through the entire membrane.

In this work, we calculated the PMF profiles for the interaction of the homologous series of 7-nitrobenz-2-oxa-1,3-diazol-4-yl (NBD)-labeled fatty amines (NBD-Cn, *n* = 4 to 16) in lipid bilayers with different compositions. The NBD-Cn homologous series has been previously characterized experimentally [7,56] and by MD simulations [57,58,59]. Since the difference between the NBD-Cn molecules is only on the size of their alkyl chain, they can be used to assess the effect of hydrophobicity on the rate of passive permeation of amphiphiles and drug-like molecules. We consider the simulations in cholesterol-containing and cholesterol-free membranes regarding the possibility to characterize the permeation process in lipid bilayers that may emulate several biological membranes, namely the apical and the basolateral sides of the plasma membrane and the membranes of internal organelles. In order to keep simplicity, considering the absence of formal charge on the amphiphiles studied in this work, we mimic the outer leaflet with palmitoyl sphingomyelin (SpM):Chol (6:4) and the inner leaflet with 1-palmitoyl-2-oleoyl-*sn*-glycero-3-phosphocholine (POPC):Chol (1:1). Those two lipid compositions are first studied as independent and symmetric bilayer systems. As a matter of comparison, the effects of membrane asymmetry were also assessed through independent simulations in a SpM:Chol (6:4)/POPC:Chol (1:1) asymmetric bilayer. To gain predictive power for the permeation of amphiphilic drugs, the obtained parameters must be understood in terms of the established interactions. This was achieved through the calculation of the PMFs and through the detailed analysis of each individual simulation where the amphiphile is restrained at a given bilayer depth.

## 2. Materials and Methods

### 2.1. Unrestrained MD Simulations for Membrane Characterization

Data concerning unrestrained simulations in symmetric POPC membranes were obtained from our previous study [57]. For the simulations on POPC:Chol (1:1) and SpM:Chol (6:4) bilayers and with the asymmetric bilayers, MD simulations and analysis of trajectories were carried out using the GROMACS 4.5 package [60,61]. The topology of the POPC [62] based on Berger et al. [63] parameters was used, with the dihedrals around the double bond described according to [64], which is reported to be more appropriate for cholesterol-containing bilayers. The topology of the SpM molecule [65] was downloaded from Lipidbook [66]. For cholesterol, we used the description of Holtje et al. [67]. While this force field has been put into question [68], we have applied it successfully in the past to the simulation of NBD-Cn in lipid bilayers [57,58]. Therefore, for the sake of consistency and comparison with other previous works, it was chosen for this study. The POPC:Chol (1:1) bilayer is composed of 144 lipids hydrated by 5824 water molecules, and the SpM:Chol (6:4) is composed of 150 lipids hydrated by 6612 water molecules. The SPC water model [69] was used. An asymmetric SpM:Chol (6:4)/POPC:Chol (1:1) bilayer was built by pasting a SpM:Chol (6:4) monolayer on the top of a POPC:Chol (1:1) monolayer, leading to a system composed by 45 SpM and 30 Chol molecules in the top monolayer and 37 POPC and 37 Chol molecules in the bottom monolayer. Molecular structures are shown in Figure S1.

After energy minimization and equilibration steps identical to those described in reference [59] for symmetric bilayers, production MD simulations of asymmetric membranes were carried out for 100 ns, using a 2 fs integration step. These runs were carried out under a constant number of particles, pressure (1 bar) and temperature (298.15 K), and with periodic boundary conditions. Pressure and temperature control were carried out using the Berendsen [70] and V-rescale [71] schemes, with coupling times of 1.0 ps and 0.1 ps, respectively. Semi-isotropic pressure coupling was used. Van der Waals and Coulomb interactions were cut off at 1.0 nm, whereas for long-range electrostatics the Particle Mesh Ewald treatment [72] was applied. All bonds were constrained, using the SETTLE algorithm [73] for water, and the LINCS algorithm [74] for all the others.

The instant average area per lipid molecule, *a*, was calculated as the instant box area divided by the number of lipid molecules in each monolayer, for both pure lipid bilayers and NBD-containing bilayers. The surface areas of each type of molecule composing each bilayer, POPC:Chol (1:1) and SpM:Chol (6:4), were calculated as described in [75]:

(1) $$A_{PL}=\frac{2A_{box}}{V_{box}-N_{W}V_{W}}\left[\frac{V_{box}-N_{W}V_{W}-xN_{lipid}V_{Chol}}{\left(1-x\right)N_{lipid}}\right]\;A_{Chol}=\frac{2A_{box}V_{Chol}}{V_{box}-N_{W}V_{W}}$$

In these equations, *A_PL_* is the cross-sectional area per phospholipid molecule, *A_Chol_* is the cross-sectional area per Chol molecule, *A_box_* is the area of *xy* plane of the simulation box, *V_box_* is the total volume of the simulation box, *N_W_* is the number of water molecules, *V_W_* is the volume of the water molecule (0.0312 nm^3^) [75], *x* is the Chol mole fraction, *N_lipid_* is the number of lipid molecules and *V_Chol_* is the volume of the Chol molecule (0.593 nm^3^) [75]. The characterization of the lipid membranes is summarized in Table S1.

Deuterium order parameters, *S*_*CD*_, were calculated using

(2) $$S_{CD}=\frac{1}{2}3cos^{2}\theta_{i}-1$$

where *θ_i_* is the angle between a C–D bond and the bilayer normal, and the brackets denote averaging over time and C–D bonds [76]. In our simulations, using a united atom force field, deuterium positions of acyl chain atoms were constructed from the neighbouring carbon atoms assuming ideal geometries. The characterization of the order parameters is summarized in Figure S2.

### 2.2. Calculation of Free Energy Profiles of Long Amphiphiles Interacting with Lipid Membranes

Data concerning the simulations in symmetric POPC bilayers for the calculation of free energy profiles are taken from our previous study [58], and we refer the reader to this reference for details. The conditions of the umbrella sampling simulations are identical to those described below for the cholesterol-containing bilayers. Sampling simulations were run for 100 ns, where the first 20 ns of each simulation were used for equilibration and the last 80 ns for analysis.

The corresponding simulations in Chol-containing bilayers were carried out in GROMACS 4.5, with the same basic force field, integration step, ensemble, boundary conditions, constraints, pressure and temperature control and cut-off scheme choices as used in their unrestrained counterparts.

#### 2.2.1. Umbrella Sampling Simulations in Cholesterol-Containing Bilayers

The distance of the NBD center of mass (COM) to the membrane COM along the normal coordinate *z* was chosen as the reaction coordinates for solute permeation, where *z* = 0 nm is defined by the COM of the lipid molecules. The simulations were carried out according to the pull geometry cylinder (PGC) method as described before [58], with a cylinder of radius 15 Å. Here, a weight of 1 was assigned to all atoms within a distance of 10 Å to the cylinder axis, and the weights were switched to 0 between 10 and 15 Å.

The initial structures for each umbrella window were generated by pulling the amphiphile NBD-C16 from the NBD COM, moving from the bilayer center to bulk water with a pulling rate of 0.005 nm/ps and a force constant of 500 kJ mol^−1^ nm^−2^. For the other NBD-Cn molecules (*n* = 4, 8, 12), initial structures were adapted from those of NBD-C16. Adjacent umbrella windows spanned the space between the membrane center (*z* = 0) into the bulk water region, separated by 1 Å, between 3.5 and 5 nm.

A harmonic umbrella potential, acting on the COM of the NBD moiety, was applied (force constant 3000 kJ mol^−1^ nm^−2^). After the simulations were completed, the unbiased PMF was obtained using the weighted histogram analysis method [77,78].

#### 2.2.2. Convergence of Free Energy Profiles

As discussed in large detail in our previous work [58], there are two issues regarding the computation of free energy profiles. For each umbrella sampling window, how long is the simulation time required to equilibrate the system, and how long is the time required to sample the system appropriately? If the equilibration and sampling times for the given system and the given sampling windows are not sufficiently long, then the results do not converge to their true values.

As described elsewhere [58], we use three ways to consider the above problems:

(i) Assuming (incorrectly) that no equilibration is needed, in each sampling window using increasing amounts of simulation time for the sampling of the PMF.
(ii) In each sampling window, systematically increase the slice of the simulation time used for equilibration, and use the rest of the simulation data for analysis.
(iii) Again in each sampling window, systematically increase the amount of data used for equilibration, being the analysis for the PMF profile (in each window) carried out over a fixed period of 20 ns.

In all three schemes, not only the values of the barriers but also the shapes of the profiles are compared to each other. For NBD-C16, 200 ns simulations were carried out. For the other amphiphiles, 100 ns simulations were performed. The first 20 ns of each simulation were used for equilibration and the remaining time for analysis. Error bars were calculated by the difference between PMF profiles generated from the two halves of the sampling time.

#### 2.2.3. Permeability Calculations Using the Inhomogeneous Solubility-Diffusion Model (ISDM)

According to the solubility-diffusion model, permeation of molecules across membranes occurs by a three-step process involving the partition of the molecule from the aqueous phase on one side into the bilayer, its diffusion across the bilayer, and its partition from the bilayer into the aqueous phase on the other side. This model may be improved by considering different regions *z* of the bilayer normal with a specific partition coefficient and different diffusion coefficients within each region. At a microscopic detail, the overall membrane resistance *R* to solute permeation, defined as the inverse of the permeability coefficient *P*, can be expressed as the integral over the local resistances across the membrane,

(3) $$R=\frac{1}{P}=\int_{0}^{d}\frac{dz}{K\left(z\right)D(z),}$$

where *K*(*z*) and *D*(*z*) are the depth-dependent partition coefficient from water into the membrane and the diffusion coefficient in the membrane at depth *z*, respectively, and *d* is the membrane thickness [79]. Using MD with special sampling techniques it is possible to calculate those equilibrium and dynamic properties related to the permeation process.

The equilibrium property is the solute partition between water and different regions in the membrane and is expressed by the free energy of solute transfer from water into various depths *z* of the membrane, ∆*G*(*z*). The solute ∆*G*(*z*) is related to its partition coefficient *K*(*z*) through:

(4) $$K\left(z\right)=exp(-\frac{\Delta G\left(z\right)}{RT}),$$

with *R* being the gas constant and *T* being the absolute temperature.

The dynamic property is the solute diffusion coefficient at position *z* in the membrane. Considering solute diffusion in a medium, the local time-dependent friction coefficient of the diffusing molecule ξ(*t*) is related to the time autocorrelation function of the fluctuations of the instantaneous *F*(*z*, *t*) from the mean 〈*F*(*z*)〉*_t_* by

(5) $$\xi \left(t\right)=\frac{\langle \Delta F\left(z,t\right)●\Delta F(z,0)\rangle }{RT},$$

where

(6) $$\Delta F\left(z,t\right)=F\left(z,t\right)-\langle F{(z)\rangle }_{t}.$$

Assuming that ξ(*t*) is large and decays rapidly compared to other time scales in the system, a satisfactory description of the full dynamics is provided by the static friction coefficient ξ:

(7) $$\xi =\int_{0}^{\infty }\xi \left(t\right)dt=\int_{0}^{\infty }\frac{\langle \Delta F\left(z,t\right)●\Delta F\left(z,0\rangle \right)}{RT}dt.$$

When studying diffusion across a free energy barrier, the above condition is met if the slope of the free energy barrier over a distance covered by the particle during the decay time of its friction coefficient is lower than the thermal fluctuation, *RT*. In this case, ξ is related to the local diffusion coefficient *D*(*z*) of the permeating solute at depth *z* by

(8) $$D\left(z\right)=\frac{RT}{\xi }=\frac{{\left(RT\right)}^{2}}{\int_{0}^{\infty }\langle \Delta F\left(z,t\right)●\Delta F\left(z,0\right)\rangle dt}.$$

Calculating ∆*G*(*z*) and *D*(*z*), the overall resistance *R* to permeation is obtained by integrating over the local resistances *R*(*z*) at different depths in the membrane, and the permeability coefficient *P* of the solute is obtained as the inverse of *R*:

(9) $$R=\int_{outside}^{z}ℜ\left(z’\right)dz\prime =\int_{outside}^{z}\frac{exp\left(\Delta G\left(z\prime \right)/RT\right)}{D\left(z\prime \right)}dz\prime =\frac{1}{P}.$$

The ISDM has been applied to several hydrophilic and hydrophobic molecules calculating the local resistance of the membrane to the permeation as the inverse of the local permeability coefficient. It was found that for hydrophilic molecules the main barrier is represented by the hydrocarbon core, while for hydrophobic molecules, the main barrier to permeation is offered by the head group region [50,52,54].

## 3. Results and Discussion

### 3.1. PMF Profiles for the NBD-Cn Homologous Series through Lipid Membranes

**Figure 1:**
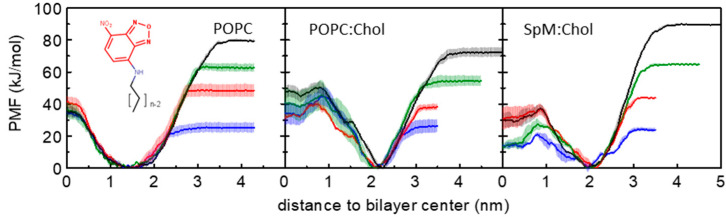
PMF profiles of NBD-C4 (blue), NBD-C8 (red), NBD-C12 (green) and NBD-C16 (black) in POPC, POPC:Chol (1:1) and SpM:Chol (6:4) bilayers, as indicated in the plots. For details on PMF generation, see Section 2.2.1. Generic structure of NBD-Cn amphiphiles is shown in the left plot.

The PMF profiles allow the calculation of free energy differences (Δ*G*) between distinct positions along a reaction coordinate. The PMF profiles for the symmetric bilayers are summarized in Figure 1, showing in separate plots the PMF of all amphiphiles for each studied bilayer. For better comparison of the data, the reference values of the PMF profiles were defined at the equilibrium position (minimum energy location in the reaction coordinate). As expected from the similarities between the amphiphiles (that have the same polar fluorophore), and in accordance with our previous unrestrained MD simulations for this homologous series in POPC membranes [57] and in cholesterol-containing membranes [59], all PMF profiles have a minimum value when the NBD is located near the head group region. For distances closer to the bilayer center, the PMF profile raises due to the presence of the translocation barrier, while for distances closer to water it raises due to the transfer of the amphiphile to the water which is a poor solvent for these molecules. As shown in equilibrium unrestrained simulations, the minimum in the free energy path is located at a higher distance from the center of the bilayer for the cholesterol-containing membranes. This is not only because of the higher membrane thickness but also because the equilibrium position of the NBD group is indeed in a more external position in the membranes with cholesterol [59]. This more external position of the NBD group in the cholesterol-containing membranes may be related to a larger lateral pressure at the membrane/water interface and with the alignment of the NBD dipole moment with the larger dipole potential in the POPC:Chol membranes [80]. The larger dipole potential of the cholesterol-enriched membranes increases the relative contribution of the interaction between the dipoles and leads to the displacement of the NBD group towards a more external position in the membrane where the interaction between the dipoles becomes attractive. An interesting feature of the PMF profiles in the membranes with cholesterol is the existence of a local minimum at the center of the bilayer. Although the presence of the local minimum was at first quite unexpected, it is indeed present in the PMF profiles of several solutes in cholesterol-containing membranes [27,81,82]. Moreover, it has been shown experimentally that cholesterol accumulates in the middle of 1,2-diarachidoyl-*sn*-glycero-3-phosphocholine (DAPC) bilayers with high cholesterol fractions [83,84]. However, we should note the considerable difference between the lipid bilayers used in this study compared to DAPC and the different structures of the NBD-Cn amphiphiles and cholesterol. The existence of a local minimum at the bilayer midplane may be justified by the low density in that region of the membrane, which in the cholesterol-containing membranes has an accentuated decrease compared to pure phospholipid bilayers [57,59].

**Figure 2:**
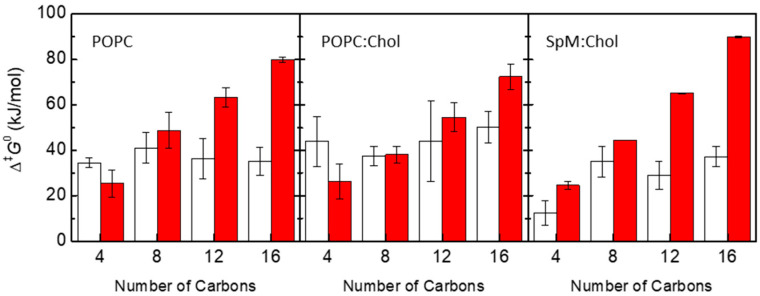
Energy barriers (Δ^‡^*G*^0^) for the processes of translocation (white) and desorption (red) of each amphiphile in POPC, POPC:Chol (1:1) and SpM:Chol (6:4) bilayers, as indicated in the plots.

Figure 2 shows the energy barriers for the processes of desorption and translocation of each amphiphile in each symmetrical bilayer. The energy barrier for the desorption increases linearly with the number of carbons as expected for interactions dominated by the hydrophobic effect. On the other hand, there is no clear tendency regarding the dependence of the translocation energy barrier on the number of carbons, both for pure POPC and cholesterol-containing bilayers. Variations in the height of the energy barriers at the bilayer center along the homologous series seem to be caused by possible sampling issues in the cholesterol-containing systems. The dependence of energy barriers, for both desorption and translocation processes, on the number of carbons of the amphiphile alkyl chain is in agreement with experimental results for the transfer of labeled phospholipids between vesicles [5,85], and with the kinetics of interaction of these amphiphiles with POPC bilayers [7]. For long-chain amphiphiles NBD-C12 and NBD-C16, the energy barrier for desorption is higher than that for translocation. In opposition, for NBD-C4 the energy barrier for translocation is higher than for desorption, except in the SpM:Chol bilayer. For phospholipids and long-chain amphiphiles that fit the full length of the lipid monolayer, the height of the energy barrier for translocation is expected to be directly related to the energy required to place the polar groups in the nonpolar center of the bilayer. Therefore, in homologous series where the polar part of the molecule is maintained, one would expect that the energy barrier for translocation is not strongly dependent on the nonpolar part of the molecule. This is in fact observed for this homologous series in the POPC and POPC:Chol membranes (Figure 2, left and middle panels), and observed experimentally for POPC membranes [7]. However, for very ordered membranes, the presence of solute molecules that do not fit the length of the lipid monolayer will cause membrane perturbations that may contribute to a faster translocation process [86,87]. This could be the reason for the significantly lower energy barrier observed for NBD-C4 in SpM:Chol membranes (Figure 2, right panel).

The latter argument also applies to the interpretation of the translocation energy barriers in cholesterol-containing and cholesterol-free lipid bilayers. In general, the translocation rate constants measured experimentally for phospholipids are smaller in the membranes with cholesterol than in POPC pure bilayers [9]. This idea has been also reported in MD studies addressing different lipid bilayers [88]. However, in the case of the NBD-Cn amphiphiles, this is not evident from the height of the obtained translocation energy barriers shown in the plots of Figure 2. A feature contributing to a slower translocation in cholesterol-containing bilayers may be related to the wider free energy barrier in membranes with cholesterol. Considering the model of diffusion through the barrier [29,89,90], this would lead to a considerable decrease in the translocation rate constant. However, a definite interpretation of the free energy profiles requires experimental data for the kinetics of these NBD-Cn amphiphiles in cholesterol-containing membranes, complementing the existing data in simple POPC bilayers reported by our group [7,56].

**Figure 3:**
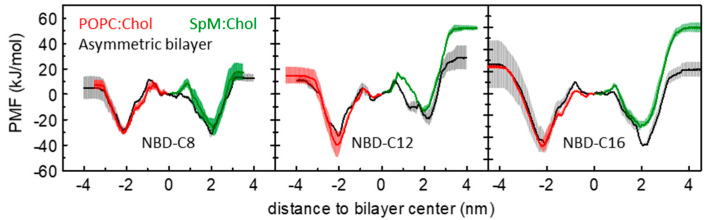
Comparison between the PMF profiles obtained for the POPC:Chol (1:1) (red) and SpM:Chol (6:4) (green) and the PMF profiles obtained for the asymmetric bilayer (black). Data is shown for NBD-C8 (**left**), NBD-C12 (**middle**) and NBD-C16 (**right**).

We addressed the effect of asymmetry of lipid bilayers in the interaction with the amphiphilic molecules. Figure 3 compares the calculated PMF profiles for these amphiphiles in symmetrical and asymmetrical systems. This representation assumes the reference of the PMF in the bilayer center, for better comparison between the profiles in the symmetric systems with the transmembrane profile obtained for the asymmetric system. The PMF obtained in the POPC:Chol leaflet of the asymmetric membrane is very similar to that obtained in the symmetric membrane (PMFs in red and black respectively). However, for SpM:Chol membranes, although the profiles are not qualitatively dissimilar, the free energy barrier for desorption from the SpM:Chol side in the asymmetrical bilayers seems consistently lower than that determined in the corresponding symmetrical SpM:Chol systems, especially for the longer-chained NBD-C12 and NBD-C16 amphiphiles. For the latter two molecules, the free energy profiles from the center to water through the two different leaflets in the asymmetric membrane are considerably more similar than could be anticipated from the curves obtained in the symmetric systems. This suggests that the presence of the less ordered POPC:Chol leaflet influences the properties of a SpM:Chol leaflet, reflecting some degree of coupling between the two leaflets [91,92,93,94,95]. However, the effects are relatively small, and the high rugosity observed in the PMFs obtained in the asymmetric bilayer suggests that the differences may also reflect sampling issues. As described in [58], sampling problems can preclude a rigorous quantitative evaluation of the results, and their qualitative interpretation is much more straightforward. Considering methodological developments on [58], here all results are given by simulations using the PGC (pull geometry cylinder) scheme, as described in Section 2.2.1. The convergence of the free energy barriers for all amphiphiles, in POPC:Chol (1:1), SpM:Chol (6:4) and POPC:Chol (1:1)/SpM:Chol (6:4) is shown in Supplementary Materials, Figures S3–S5. From the above, in the following, we will focus on the symmetric lipid bilayers.

In addition to the characterization of the free energy profiles, several different properties were evaluated from the trajectories of all performed simulations, including variations in the orientation and H-bonding of the polar NBD group, and alkyl chain conformation with the transverse location of the amphiphile. The analysis of important system properties at different positions along the reaction coordinate (Figures S6–S14) gives an interesting description of bilayers and amphiphiles during the permeation process. Pure POPC, in the liquid-disordered state, is more prone to deformations than the cholesterol-containing membranes (Figure S6). Regarding the amphiphiles, the maximal value of the distance between the head group and the terminal carbon (Cter) of the alkyl chain is attained just before desorption and correlates well with a situation close to an *all-trans* conformation (Figure S7). However, regardless of the environment, the alkyl chains have a preference for a stretched conformation, even when in the water (Figures S8–S10). Regarding H-bonding interaction, as expected, as the NBD approaches/inserts into the bilayer, the probability of H-bonding to the lipid suddenly increases (Figure S11 left plots). One would expect that as the NBD group goes deeper into the membrane, the interaction with lipid polar groups would vanish. This is in fact observed in the cholesterol-containing membranes where no H-bonds are formed for *z* ≤ 1.5 nm. However, for POPC-only membranes H-bonds are maintained down to *z* = 0.5 nm. Regarding the interaction of the NBD group with water molecules, the frequency of H-bonding decreases as the NBD group inserts in the membrane, as expected. A relatively high probability of establishing H-bonds with water when the NBD is close to the bilayer center is also observed for the POPC bilayers but not for those containing cholesterol (Figure S11 middle and right plots). The different behaviour observed in the POPC membrane is a consequence of its higher deformability, where the polar lipid headgroup and water molecules can follow the NBD group. This is also reflected in a decrease in the P-P distance when the NBD is located at around 1 nm from the bilayer center (Figure S6). 

**Figure 4:**
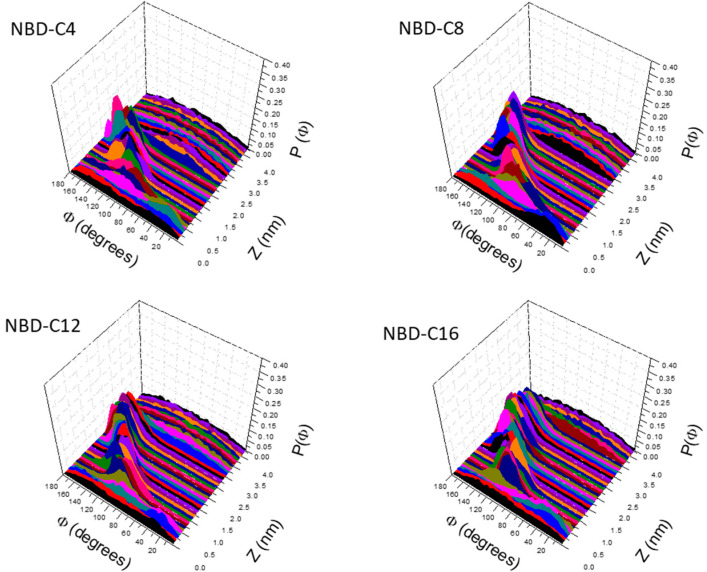
Orientation of the short axis of the NBD group (see definition in Figure S1) at different bilayer depths in POPC bilayers for the studied NBD-Cn amphiphiles.

The orientation of the NBD group when located at different depths in the POPC bilayer is shown in Figure 4. When in bulk water (*z* ≥ 3 nm for NBD-C4) no preferential orientation is observed. When the amphiphile inserts its alkyl chain in the lipid bilayer, a strong preference for orientations close to 180° is observed. In this orientation the NO_2_ group is pointing towards the aqueous medium, allowing the alkyl chain to orient parallel to the lipids in the bilayer. The NBD group has a large dipole moment (9 D), oriented with the negative pole in the NO_2_ group and the positive pole in the alkyl chain [96]. Thus, when inserted in the lipid bilayer at the equilibrium position, the dipole moment of the NBD amphiphiles is oriented parallel to the dipole potential of the membrane, in a repulsive interaction. This orientation is attained to allow the alignment of the amphiphilic moment of NBD-Cn and that of the lipids, with the alkyl chain of NBD-Cn inserted deeper and aligned with the lipid acyl chains. Accordingly, the angle distribution is broader for NBD-C4 (with a small alkyl chain) and is more accentuated for the amphiphiles with higher amphiphilic moments. As the NBD group is moved from the equilibrium position towards the center of the membrane, a shift is observed towards the attractive antiparallel orientation between the amphiphile dipole moment and the membrane dipole potential. However, exactly in the center of the bilayers, the orientation of the NBD group is mostly random. This shows that at this position, the orientation of the alkyl group parallel to the bilayer surface (not inserting in any of the leaflets, Figure S7 right plots) is the major energetic requirement.

Similar results are obtained for the orientation of the NBD group in the cholesterol-enriched membranes. The major difference is that deviations from the orientation parallel to the bilayer normal are observed only very close to the bilayer center. This shows that in more ordered membranes, the alignment of the amphiphilic moments dominates (Figures S13 and S14).

### 3.2. Quantitative Evaluation of Permeation of the Amphiphiles through the Lipid Bilayers—Application of the Inhomogeneous Solubility-Diffusion Model

The data obtained from MD simulations allow the calculation of permeability coefficients through the lipid membranes. This can be achieved with the application of the ISDM [79], and has been applied to the permeation of small and large molecules [50,52,54,97]. To apply this model, as described in Section 2.2.3, the PMF and the local diffusion coefficient through the lipid membrane are needed. Then, the local resistance to the permeation and the overall permeability coefficient may be obtained, Equations (3)–(9).

**Figure 5:**
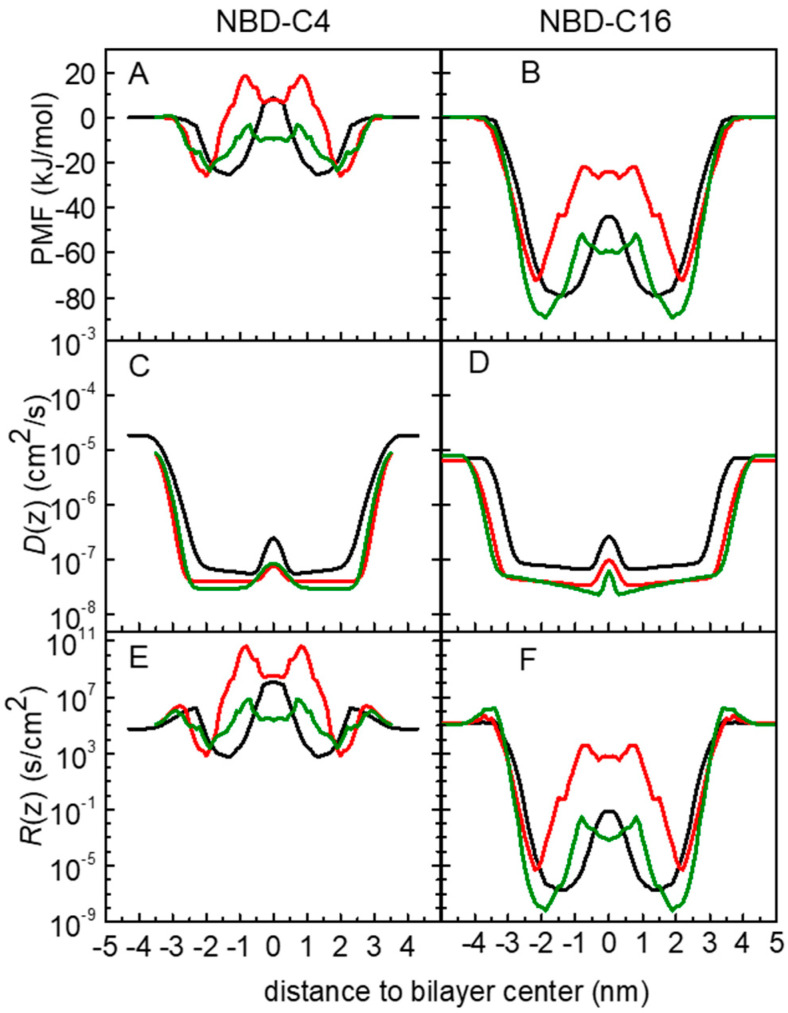
Free energy (**A**,**B**), local diffusion coefficient (**C**,**D**) and local resistance (**E**,**F**) profiles for the amphiphiles NBD-C4 (**A**,**C**,**E**) and NBD-C16 (**B**,**D**,**F**) in POPC (black), POPC:Chol (1:1) (red) and SpM:Chol (6:4) (green) bilayers.

In Figure 5, we present the free energy, local diffusion coefficient and local resistance profiles for the amphiphiles, NBD-C4 and -C16 in the symmetric POPC, POPC:Chol and SpM:Chol bilayers. Data for all amphiphiles are shown in Figure S15. The PMF profiles were already discussed in the previous sections. Regarding the local diffusion coefficients, *D*(*z*), the profiles obtained are in accordance with those expected for molecules embedded in a lipid bilayer [54]. Generally, when the molecules are in the water, *D*(*z*) has values in the order of 10^−5^ cm^2^/s as expected. As the molecules start to interact with the lipid bilayer, *D*(*z*) decreases and in general reaches a minimum at the high-density region of the bilayer. At the bilayer midplane, *D*(*z*) increases again due to the lower density of the membrane in that region. 

The performed analysis allowed us to obtain the resistance profile, *R*(*z*), of the amphiphiles through the lipid bilayers. As described in Section 2.2.3, the permeability coefficient is calculated as the inverse of the resistance to the permeability, obtained as an integration of the local resistances across the membrane. The resistance profiles obtained show a common pattern for all amphiphiles. In accordance with the ISDM equation, for equally spaced sampling windows, the resistance profile depends on the PMF and on the *D*(*z*) profiles. Starting at the pure water phase, as the NBD approaches the membrane, there is a small resistance barrier. This is mainly governed by a decrease in the local diffusion coefficient as the amphiphiles start to interact with the bilayer in a region where the PMF profile is still flat. This small resistance barrier agrees with the experimental observation that insertion in the membrane is not diffusion-controlled [4,5,6,7,10].

A minimum in the resistance profile is achieved at the equilibrium position of the amphiphile (roughly at the same depth as the PMF minimum). At deeper locations, the resistance rises again, with a local maximum near the bilayer center (absolute for NBD-C4). Since *D*(*z*) does not change significantly in this region of the membrane, the shape of the resistance profile is mainly governed by the PMF profile. Reflecting the symmetry of the bilayer, the resistance decreases again to a minimum at the equilibrium position in the other leaflet and finally shows another barrier when desorbing from the membrane into the water phase. In agreement with the PMF profiles, in the cholesterol-containing bilayers the resistance profiles also show a local minimum in the bilayer midplane.

The application of the ISDM has been discussed in several works. It has been reported that this model may overestimate the permeability of hydrophobic molecules, for which the rate-limiting step for permeation is the desorption instead of the translocation energy barrier. [98,99] This limitation of ISDM comes from the fact the predicted permeability coefficients are the result of the inverse of the integral of the exponential of the free energy profile, where positive Δ*G*(*z*) values will strongly contribute to decreasing the permeability, while negative Δ*G*(*z*) will almost not affect it. In our study this is expected for the longer chain derivatives, such as NBD-C16, reflecting the low resistance values within the bilayer, as illustrated in Figure 5F. 

**Figure 6:**
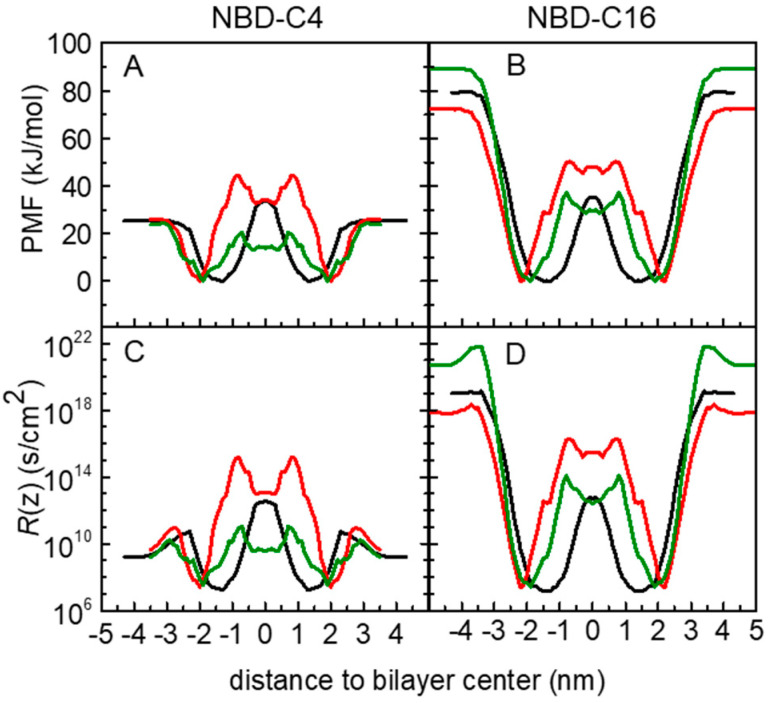
Free energy (**A**,**B**), and local resistance (**C**,**D**) profiles for the amphiphiles NBD-C4 (**A**,**C**) and NBD-C16 (**B**,**D**) in POPC (black), POPC:Chol (1:1) (red) and SpM:Chol (6:4) (green) bilayers, taking the equilibrium position of the amphiphiles as reference for the PMF profiles.

Different strategies have been used to overcome this limitation of ISDM when applied to hydrophobic molecules. These include the “permeability predictor” considering only the Δ*G*_max_ and Δ*G*_min_ values of the PMF [98], and more recently offsetting the PMF profiles by a free energy of solvation [99]. Both these approaches have a heuristic character, without theoretical evidence. In the present work, to keep the ISDM integration of the resistance profile, avoiding the addition of other energetic contributions, we adopted a different heuristic strategy: we considered the energy reference of the PMF profiles at the equilibrium position in the lipid membranes. This procedure was used by us to calculate cholesterol translocation across lipid membranes [29]. The results obtained for the resistance profiles of NBD-C4 and NBD-C16 are shown in Figure 6. Data for all amphiphiles are shown in Figure S16. As expected, this offset of the PMF profiles results in higher resistance values, with a larger impact for the more hydrophobic amphiphiles. In these cases, the desorption from the lipid membrane contributes very significantly to the overall resistance, in agreement with this being the rate-limiting step for the permeation [7,11]. It should be noted that this strategy has some limitations when considering the permeation between two aqueous compartments separated by a lipid bilayer. Changing the energy reference results in the offsetting of the PMF profile. Consequently, this also offsets the resistance values on the entire permeation path, including the water phase region. This effect has two main problems. Molecules with similar water diffusion coefficients would end up with different resistance values in water at the beginning of the permeation process. Additionally, the more hydrophobic amphiphiles would be accounted with larger resistance to insert in the lipid bilayer. Although a decrease in the rate constant for insertion is in fact observed with the increase in the amphiphile hydrophobicity [4,5,7,10], its magnitude is lower than predicted by offsetting the PMF profiles. Despite this limitation, we will proceed with the analysis of both approaches, the direct application of ISDM (PMF reference in the water) and considering the PMF reference in the energy minimum.

**Figure 7:**
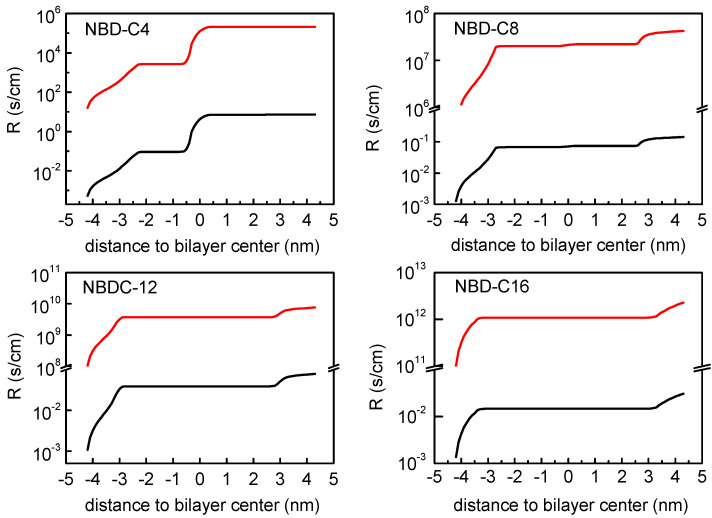
Integration of the profile of the local resistances across the lipid bilayer of POPC, for NBD-C4, NBD-C8, NBD-C12 and NBD-C16. Resistance profiles assuming the ISDM (black) and a modified ISDM version assuming the equilibrium position of the amphiphiles as reference for the PMF profiles (red) are shown. Note the logarithmic representation and the break in the scale of the resistance values.

The membrane regions that contribute the most to the resistance to permeation may be clearly evaluated plotting the integration of the resistances across the lipid bilayers as shown in Figure 7. Three main regions contributing to this resistance are identified: the entrance to the membrane, the transition through the bilayer midplane, and finally the desorption from the bilayer. The relative contribution of each region depends on the amphiphile. For the cases where the energy barrier for the translocation is higher than that for desorption, such as shown for NBD-C4, the center of the bilayer is the region that contributes the most to the overall resistance. For the other cases (notably for the amphiphiles with longer alkyl chains), the translocation energy barrier is lower than that for desorption, and the overall resistance is determined only by the water/lipid interfaces. This is in agreement with the observation that desorption from the membrane is the rate-limiting step in the case of the NBD amphiphiles with long alkyl chains [11]. The integration of the resistance corresponding to the PMF profiles with the energy reference at the equilibrium position results in higher resistance values (red curves in Figure 7), with the same shape as that obtained with the reference in water (black curves in Figure 7), which translates into lower permeability coefficients.

**Figure 8:**
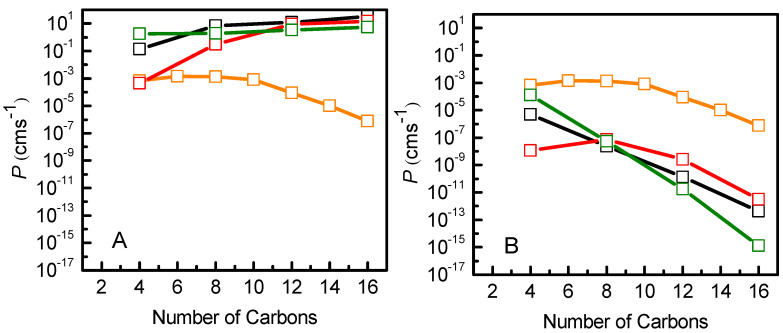
Comparison of permeability coefficients of NBD-Cn, calculated from MD simulations using the ISDM, for POPC (black), POPC:Chol (1:1) (red) and SpM:Chol (6:4) (green), and calculated from the integration of the kinetic rate constants through a cell monolayer mimicked by POPC [11] (orange). The application of the ISDM considers the origin of the PMF profiles in the water (**A**) or in the energy minimum of the system (**B**).

Finally, we used the resistance data to calculate the permeability coefficients for the NBD-Cn molecules. In Figure 8 the permeability coefficients from the MD data considering the zero of the PMF profile either in the water (Figure 8A) or in the minimum energy position (Figure 8B) are shown, in comparison with the permeability coefficient through a cell monolayer mimicked by POPC [11], calculated from experimental data [7]. The direct application of the ISDM predicts an increase in the permeability coefficient with the length of the alkyl chain in contradiction with experimental results. On the other hand, the introduced modification on the PMF profiles used in the ISDM allows the correct qualitative evaluation of the desorption as a rate-limiting step in the permeation process, showing smaller permeation coefficients for longer chain amphiphiles as a result of their lower desorption rate constants [7]. Thus, considering the zero of the PMF in the minimum energy position, it is possible to qualitatively describe the regimes where the translocation or the desorption are the rate-limiting steps (Figure 8B). While quantitative comparisons with experiments and/or results from kinetic modeling are still challenging, it is expected that increasingly improved enhanced sampling strategies will contribute to bridging this gap [100].

## 4. Conclusions

In this work, we describe the interaction of the homologous series of NBD-Cn amphiphiles with lipid bilayers of different compositions, including an asymmetric bilayer. This is a step forward towards the establishment of rules to predict the rate of passive permeation of amphiphiles and drug-like molecules. For all studied bilayers, PMF profiles have a minimum value when the NBD is near the head group region, which is more external in cholesterol-containing bilayers in agreement with our previous work [59]. The lack of evidence for systematic effects caused by membrane asymmetry tested in this work supports the approach of experimental studies in symmetric membranes mimicking each leaflet of biological membranes as reasonable approximations for transverse membrane asymmetry, at least for simple membrane compositions.

The direct comparison between experimental and calculated free energy barriers is not straightforward. Qualitatively, the energy barrier for the desorption increases linearly with the number of carbons on the NBD-Cn hydrophobic chain, on all tested bilayers. On the other hand, there is no clear tendency regarding the dependence of the translocation energy barrier on the size of the NBD-Cn chain.

The permeation of the NBD-Cn amphiphiles was evaluated by the direct application of the ISDM, resulting in higher permeability coefficients for more hydrophobic amphiphiles, contrasting with evidence from kinetic modeling based on experimental data. However, if the reference state for the PMF profile is set at the equilibrium position, a qualitative agreement is obtained. It must be stressed that in spite of the improved agreement, this approach still carries severe limitations such as an unrealistic increase in the solute resistance in the water phase. This result, together with other recent studies, highlights the shortcomings of direct application of the ISDM formalism and calls for a readdressing of theoretical approaches to calculate permeability coefficients from MD simulation data.

## Data Availability

All necessary input files may be obtained upon request.

## Supplementary Materials

The following supporting information can be downloaded at: https://www.mdpi.com/article/10.3390/membranes13060551/s1, structures and atom numbering (Figure S1); characterization of the lipid membranes (Figure S2); convergence analysis of the calculated energy barriers for cholesterol-containing systems (Figures S3–S5); analysis of several system properties during the permeation process: Membrane deformations characterized by bilayer thickness (Figure S6), distance between N1 and Cter Atoms (Figures S7–S10), hydrogen bonding (Figure S11), orientation of the NBD group (Figures S12–S14), PMF, *D*(*z*) and *R*(*z*) profiles for all amphiphiles in all bilayers (Figures S15 and S16). Areas per lipid of the components of the bilayers (Table S1).
