# Supplementary material for: Permeation of a Homologous Series of NBD-Labeled Fatty Amines through Lipid Bilayers: A Molecular Dynamics Study
Source: Membranes (Basel). 2023 May 25;13(6):551. doi: 10.3390/membranes13060551 (PMC10304540; doi:10.3390/membranes13060551)
Supplement: Supplementary file 1 [file membranes-13-00551-s001.zip › membranes-2409174-supplementary.pdf]

Supplemental Information for

# Permeation of a Homologous Series of NBD-Labeled Fatty Amines through Lipid Bilayers: a Molecular Dynamics study

**Hugo A. L. Filipe [1,2,*], Luís M. S. Loura [1,3,4] and Maria João Moreno [1,3,5]**

[1] Coimbra Chemistry Center, Institute of Molecular Sciences (CQC-IMS), University of Coimbra, 3004-535 Coimbra, Portugal; lloura@ff.uc.pt (L.M.S.L.); mmoreno@ci.uc.pt (M.J.M.)
[2] CPIRN-IPG—Center of Potential and Innovation of Natural Resources, Polytechnic Institute of Guarda, 6300-559 Guarda, Portugal
[3] CNC—Center for Neuroscience and Cell Biology, University of Coimbra, 3004-535 Coimbra, Portugal
[4] Faculty of Pharmacy, University of Coimbra, 3000-548 Coimbra, Portugal
[5] Department of Chemistry, Faculty of Sciences and Technology, University of Coimbra, 3004-535 Coimbra, Portugal
* Correspondence: hlfilipe@ipg.pt

# Contents

1. **Structures and atom numbering** 2
2. **Characterization of the Lipid Membranes** 3
3. **Convergence analysis of the calculated energy barriers for cholesterol-containing systems** 5
4. **Analysis of several system properties during the permeation process** 8
   *3.1 Membrane deformations characterized by bilayer thickness* 8
   *3.2. Distance between N1 and Cter Atoms* 9
   *3.3 Hydrogen bonding* 13
   *3.4 Orientation of the NBD group* 14
5. **Permeation of the Amphiphiles through the Lipid Bilayers** 19
   **References** 21

## 1. Structures and atom numbering

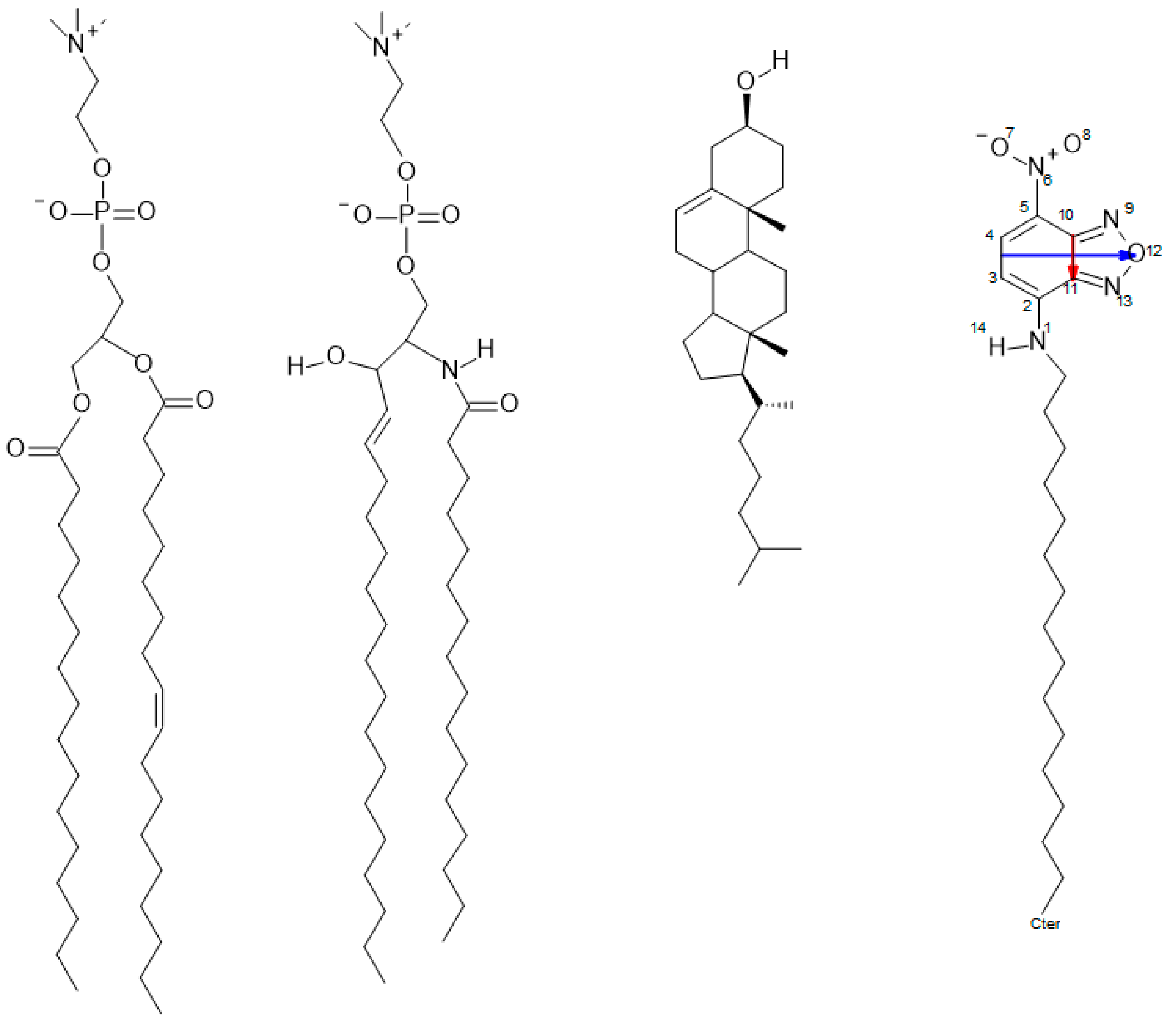


**Figure S1.** Structures of POPC, SpM, Chol and NBD-$C_{16}$ (from left to right, respectively), showing the atom numbering as used throughout the text. The blue and red arrows in NBD-$C_{16}$ denote the long and short axis (respectively) of the NBD group.

## 2. Characterization of the Lipid Membranes

This work includes POPC, POPC:Chol (1:1) and SpM:Chol (6:4) symmetric bilayers and also an asymmetric SpM:Chol (6:4)/POPC:Chol (1:1) bilayer. The area/lipid of the pure lipid bilayers is shown in Table S1, whereas the order parameter profiles of the lipidic chains are shown in Figure S2. In contrast with pure POPC, POPC in the cholesterol-containing bilayers, in the liquid ordered state, has smaller area/lipid and higher order parameters. POPC:Chol and SpM:Chol bilayers show equal average area/lipid and the maximum of the order parameter profile is also similar. Regarding the effects of the asymmetry, it does not affect the POPC:Chol leaflet significantly. However, the SpM:Chol side is slightly compressed (note the large associated error compared to the obtained difference), showing a somewhat lower area/lipid and higher order parameter than in the symmetric bilayer. This small change is not a translation of the entire order profiles, Figure S2, and therefore it should reflect small differences in the orientation of the SpM, probably not related to any effect of the membrane asymmetry itself, but due to the limited size of the asymmetric bilayer.

**Table S1.** Areas per lipid of the components of the bilayers.

| Area/Lipid (Å$^2$) | POPC | POPC:Chol (1:1) | SpM:Chol (6:4) | SpM:Chol (6:4)/ POPC:Chol (1:1) |
|---|---|---|---|---|
| **Average area** | 62.5±1.5 | 38.6±1.2 | 38.6±1.3 | 38.0±1.2/ 38.5±1.2 |
| **POPC** | 62.5±1.5 | 49.9±1.5 | - | 49.9±1.8 |
| **SpM** | - | - | 46.0±1.5 | 45.3±1.4 |
| **Chol** | - | 27.4±0.8 | 26.9±0.9 | 27.1±0.9 |

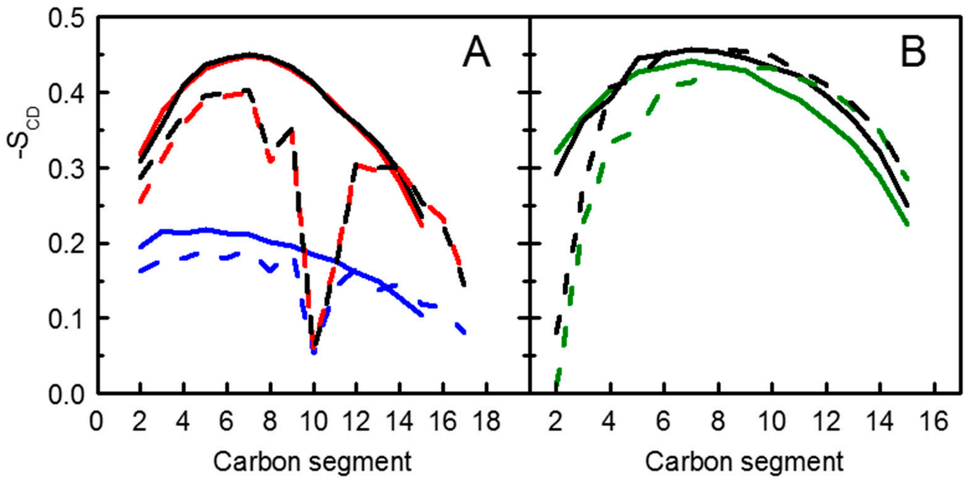


**Figure S2.** Order parameter of the POPC (**a**) and SpM (**b**) chains in the symmetric POPC (blue), POPC:Chol (1:1) (red) and SpM:Chol (6:4) (green) and of the asymmetric SpM:Chol (6:4)/POPC:Chol (1:1) (black) lipid bilayers. Solid lines refer to POPC *sn*-1 and SpM acyl chains, while dashed curves refer to POPC *sn*-2 acyl chain and SpM sphingosine.

### 3. Convergence analysis of the calculated energy barriers for cholesterol-containing systems

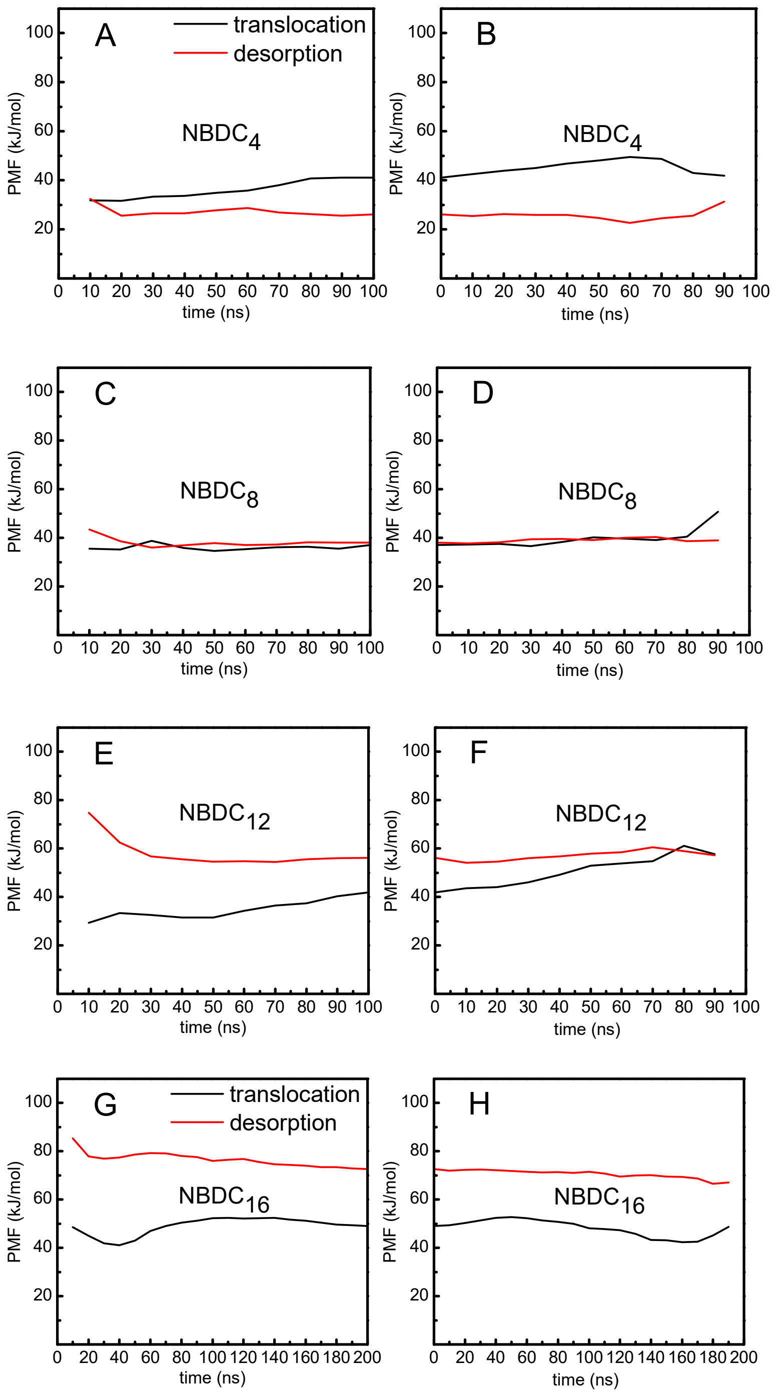


**Figure S3.** Convergence of the energy barriers for translocation (black) and desorption (red) of NBD-Cn in POPC:Chol(1:1) bilayers, calculated from the PMF profiles increasing the total simulation time (A, C, E, G) and discarding initial simulation times (B, D, F, H) for NBD-C4 (A, B), NBD-C8 (C, D), NBD-C12 (E, F) and NBD-C16 (G, H). The maximal simulation time of each umbrella window is at least 100 ns.

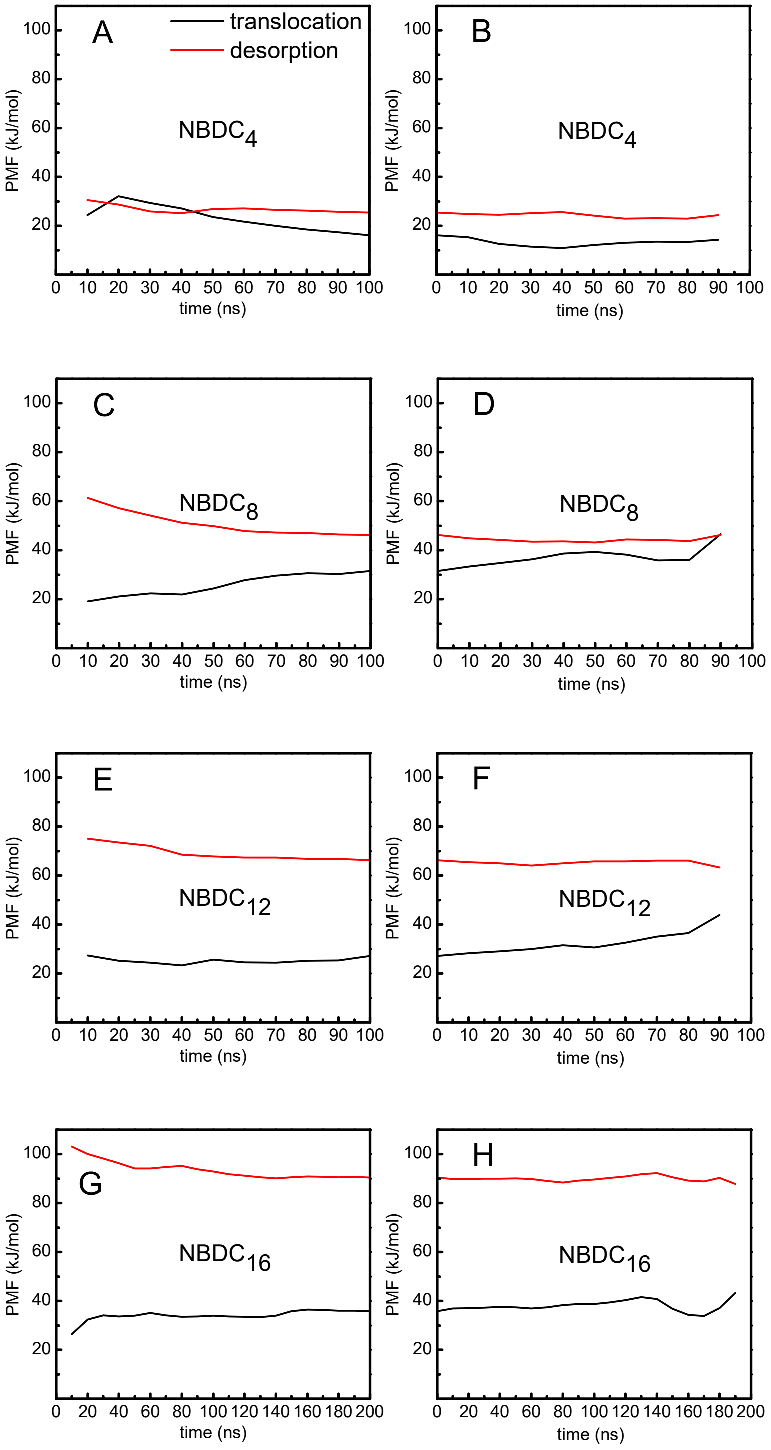


**Figure S4.** Convergence of the energy barriers for translocation (black) and desorption (red) of NBD-Cn in SpM:Chol(6:4) bilayers, calculated from the PMF profiles increasing the total simulation time (A, C, E, G) and discarding initial simulation times (B, D, F, H) for NBD-C4 (A, B), NBD-C8 (C, D), NBD-C12 (E, F) and NBD-C16 (G, H). The maximal simulation time of each umbrella window is at least 100 ns.

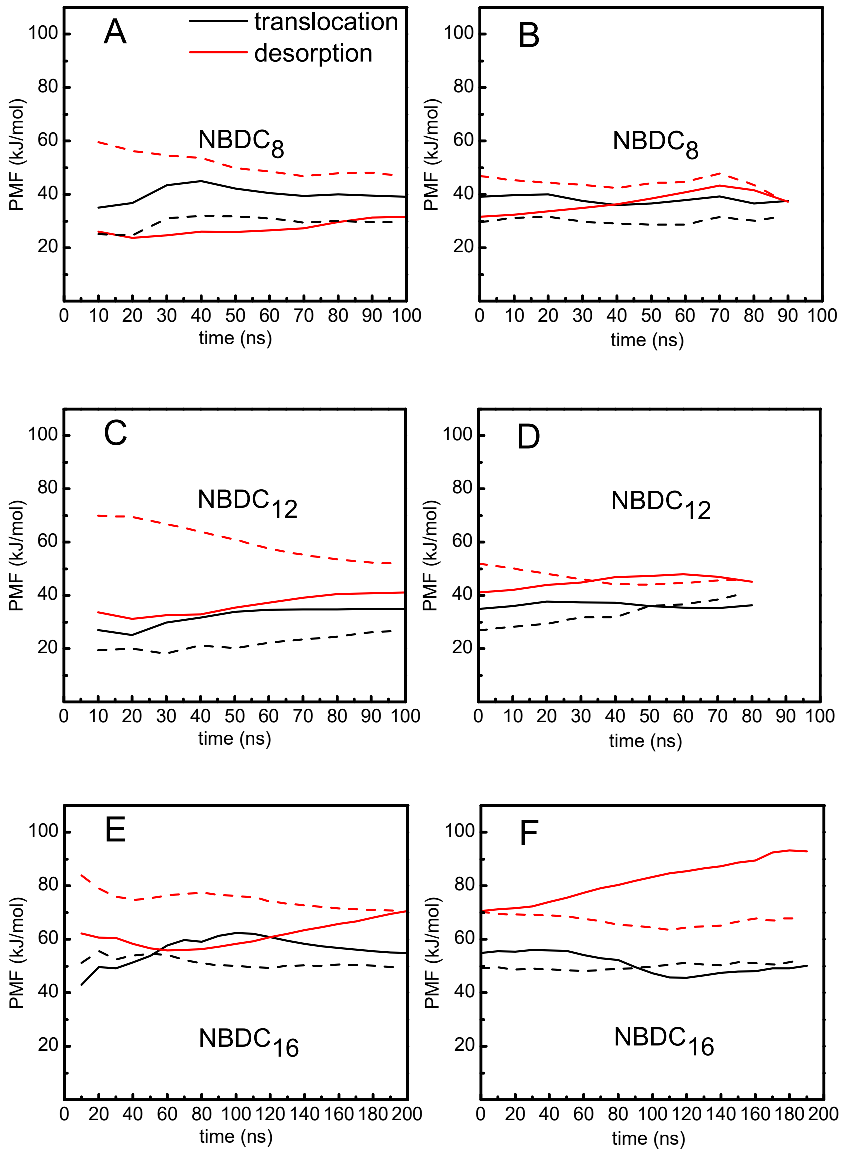


**Figure S5.** Convergence of the energy barriers for translocation (black) and desorption (red) of NBD-Cn in asymmetric bilayers for the POPC:Chol (1:1) leaflet (solid lines) and for SpM:Chol (6:4) leaflet (dashed), calculated from the PMF profiles increasing the total simulation time (A, C, E) and discarding initial simulation times (B, D, F), for NBD-C8 (A, B), NBD-C12 (C, D) and NBD-C16 (E, F). The maximal simulation time of each umbrella window is at least 100 ns.

## 4. Analysis of Several System Properties During the Permeation Process

Despite difficulties in the quantitative comparison of some simulation results with experimental data, an important feature of MD is its ability to provide atomic detail of the studied systems. In this perspective, it is possible to describe the permeation of solutes in various aspects, and obtain an illustrative view of the process. In this regard the perturbation induced in the bilayer and the behavior of the amphiphiles during the permeation process is explored here.

### *4.1. Membrane deformations characterized by bilayer thickness*

When molecules are transported to or from lipid membranes, it is quite possible that the membrane/water interface is altered due to the adsorption and desorption processes.[1] The dependence of the P-P distance on the position of the NBD group is shown in Figure S6. Regardless of fluctuations characteristic of calculating bilayer thickness values, it is quite clear that the overall bilayer thickness increases in the order POPC < POPC:Chol (1:1) < Asymmetric system < SpM:Chol (6:4). The POPC bilayer is in the liquid disordered phase and has a P-P distance below 4 nm, while the cholesterol-containing membranes, in the liquid ordered state, have P-P values on the order of 4.5 nm, being 4.6 nm for the thickest SpM:Chol membrane.

The results indicate that the pure POPC bilayer is more prone to deformations than the cholesterol-containing membranes. In pure POPC, the preferential location of the NBD moiety is at ~1.5 nm from the bilayer center. In this bilayer, the P-P distance is higher (about 3.85 nm) when the center of mass of the NBD moiety is positioned at ~2.5 nm from the bilayer center, compared to the P-P distance of ~3.8 nm when the NBD moiety is in bulk water. This reflects the protrusion of some lipids caused by the interaction with the amphiphile. For the cholesterol-containing membranes, such deformation is less evident. It seems however that when the NBD is restrained close to the bilayer midplane, the P-P distance increases, which is probably caused by the presence of an additional molecule in the membrane’s midplane.

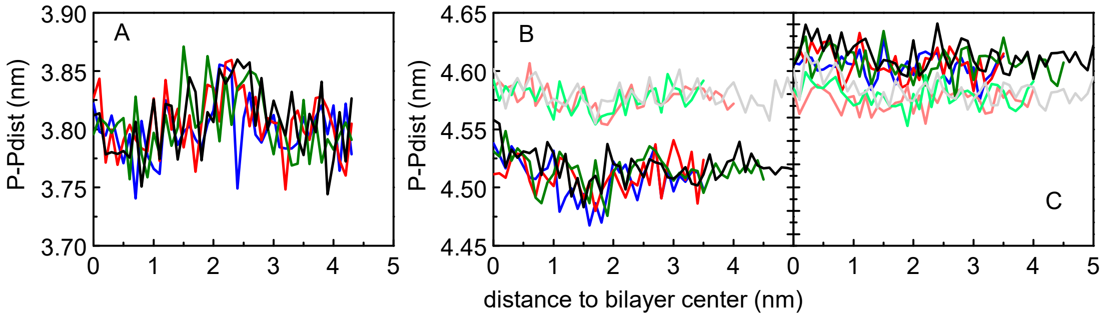


**Figure S6.** Effect on the bilayer thickness induced by the NBD-Cn amphiphiles, -$C_4$ (blue), -$C_8$ (red), -$C_{12}$ (green) and -$C_{16}$ (black), when located at different depths in the bilayer. Data for POPC (A), POPC:Chol (1:1) (B), SpM:Chol (6:4) (C) bilayers and for the asymmetric bilayer (lightened curves in B and C).

### *4.2. Distance between N1 and Cter atoms*

The distance between the N1 and Cter atoms, $\langle d \rangle_{N1\text{-}Cter}$, (see numbering in Figure S1) of the alkyl chain of the amphiphile as a function of the location *z* of the fluorophore, Figure S7 (A, B, C), gives an overall picture of the amphiphile conformation as it interacts with the membrane. The average transverse projection between N1 and Cter of each amphiphile, $\langle z \rangle_{N1\text{-}Cter}$, is shown in Figure  (D, E, F), and gives information about the orientation of the alkyl chain. The results are similar for all membranes studied. When the NBD group is close to the center of the bilayer, the distance and the *z* projection are minimal. As the distance of the NBD group from the center of the bilayer increases, $\langle d \rangle_{N1\text{-}Cter}$ and $\langle z \rangle_{N1\text{-}Cter}$ also increase, highlighting how the chain elongates and aligns with lipid acyl chains. This continues up to a point where the stretched alkyl chain abruptly leaves the bilayer, resulting in a drop of $\langle d \rangle_{N1\text{-}Cter}$ and $\langle z \rangle_{N1\text{-}Cter}$ to a value close to zero. A similar behavior was described previously for phospholipid acyl chains [2].

Some differences between cholesterol-free and cholesterol-containing bilayers may be identified. In POPC membranes, the sudden drop in $\langle d \rangle_{N1\text{-}Cter}$ and $\langle z \rangle_{N1\text{-}Cter}$ occurs at shorter distances from the center of the bilayer. This effect is due to the reduced thickness of the POPC membrane and is slightly attenuated by the membrane deformation. This difference is also attenuated by the number of insertion/desorption events of the alkyl chain in the membrane interface. In the cholesterol-containing membranes, a higher number of such events is observed. This is closely related to membrane deformation. Once inserted, the alkyl chain deforms the POPC membrane, being rarely released. In cholesterol-containing membranes the lack of deformation makes these

events more dependent on the alkyl chain movements. More insertion/desorption events contribute to softer decays in the $\langle d \rangle_{\text{N1-Cter}}$ and $\langle z \rangle$dist N1-Cter profiles.

It is also relevant to evaluate whether alkyl chains not aligned with the bilayer normal are stretched (tilted with respect to the orientation of the lipid acyl chains) or folded. This may be evaluated from the data in Figure S7, comparing $\langle d \rangle_{\text{N1-Cter}}$ (plots A, B, C) with the distance $\langle z \rangle_{\text{N1-Cter}}$ (plots E, F, G). This comparison is an overall measure of the orientation of the alkyl chain in the bilayer. It is evident that i) the alkyl chains are essentially parallel to the bilayer normal between the minimal energy position and the point of desorption, while ii) the alkyl chain samples a wide range of conformations/orientations, in the free volume pockets in the membrane, when the probe is in the center of the bilayer and in the aqueous phase.

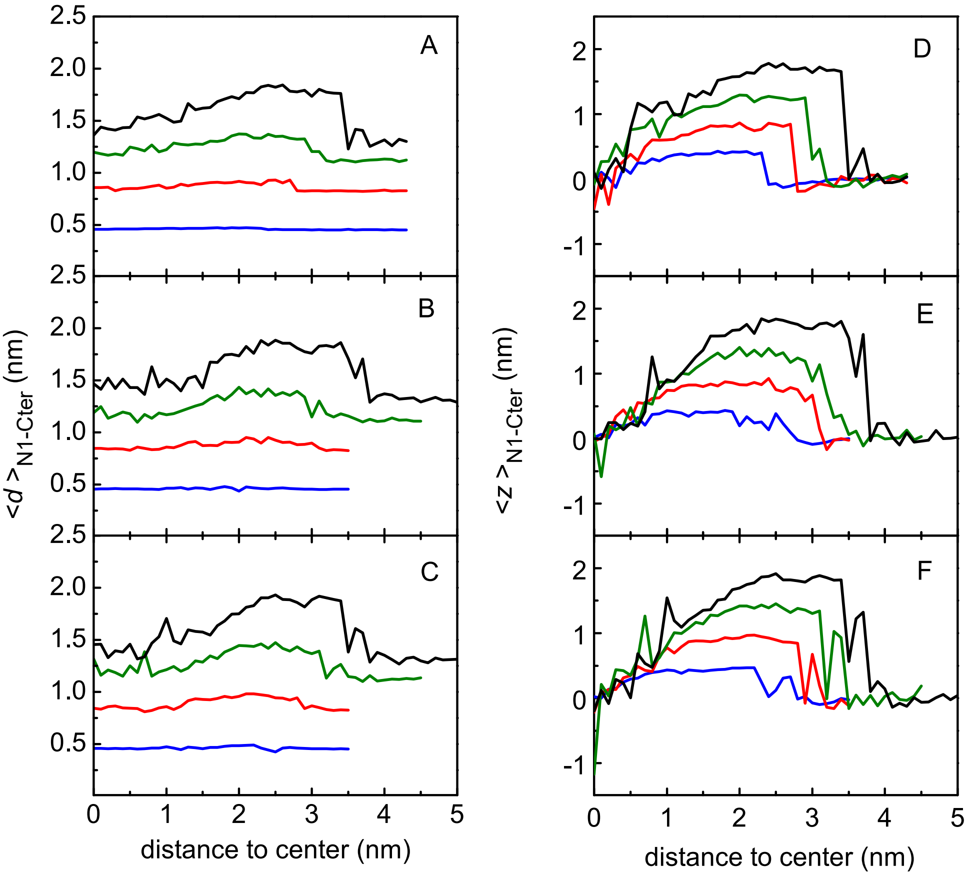


**Figure S7.** Distance, $\langle d \rangle_{\text{N1-Cter}}$ (A, B, C) and projection in the *z* axis, $\langle z \rangle_{\text{N1-Cter}}$ (D, E, F) between the atom N1 and the atom Cter of alkyl chain of the amphiphiles, NBD-C4 (blue), -C8 (red), -C12 (green) and -C16 (black), as a function of the restrained position of the NBD group in POPC (A, D), POPC:Chol (1:1) (B, E) and SpM:Chol (6:4) (C, F).

To better clarify this discussion, the distribution of the chain dihedrals was analyzed and is shown in Figures S8, S9 and S10 for POPC, POPC:Chol and SpM:Chol, respectively. According with the above discussion, the probability of *gauche* conformations (dihedral angles ~60° or ~-60°), in the case of NBD-$C_{16}$ in pure POPC membranes, is higher when the NBD is restrained close to the center of the bilayer and in the water region, Figure S8A. When the NBD is restrained close to the equilibrium position and close to the point of desorption of the alkyl chain, the number of *gauche* conformations is minimum. As shown in Figure S8B, the fraction of *trans* conformations has a maximum around 85% for all amphiphiles close to the desorption point, except for NBD-$C_4$ due to the poor anchoring of this amphiphile.

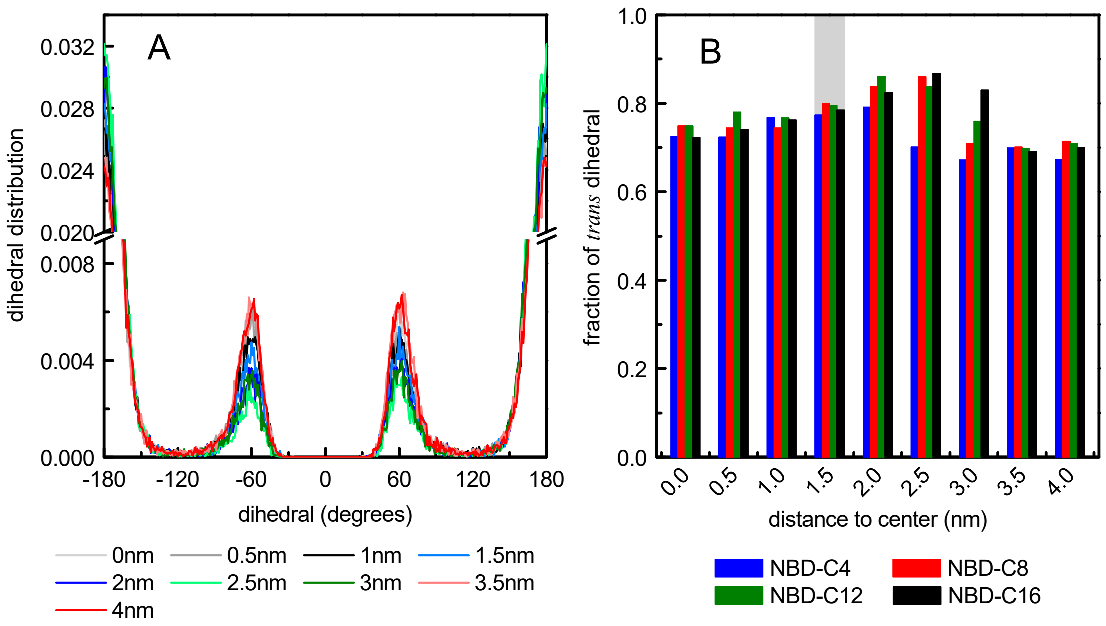


**Figure S8.** Chain dihedrals in POPC bilayers. (A) distribution of the chain dihedrals of NBD-$C_{16}$, and (B) fraction of *trans* dihedrals of the alkyl chains of the NBD-$C_n$ amphiphiles, as function of the distance of the NBD to the bilayer center. Gray bar indicates the equilibrium position of the NBD group.

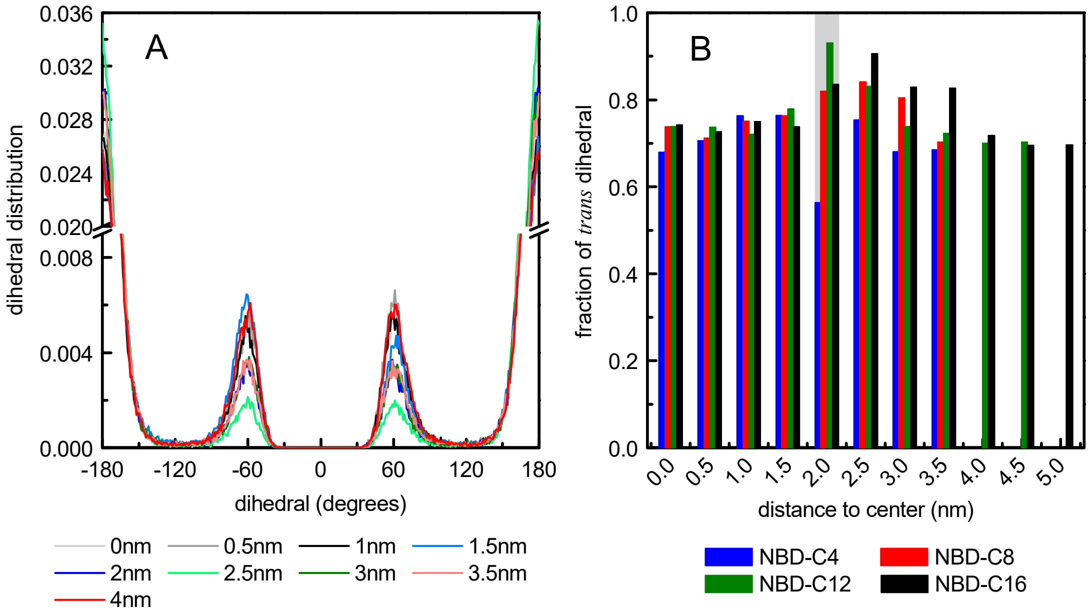


**Figure S9.** Chain dihedrals in POPC:Chol (1:1) bilayers. (A) distribution of the chain dihedrals of NBD-$C_{16}$, and (B) fraction of *trans* dihedrals of the alkyl chains of the NBD-$C_n$ amphiphiles, as function of the distance of the NBD to the bilayer center. Gray bar indicates the equilibrium position of the NBD group.

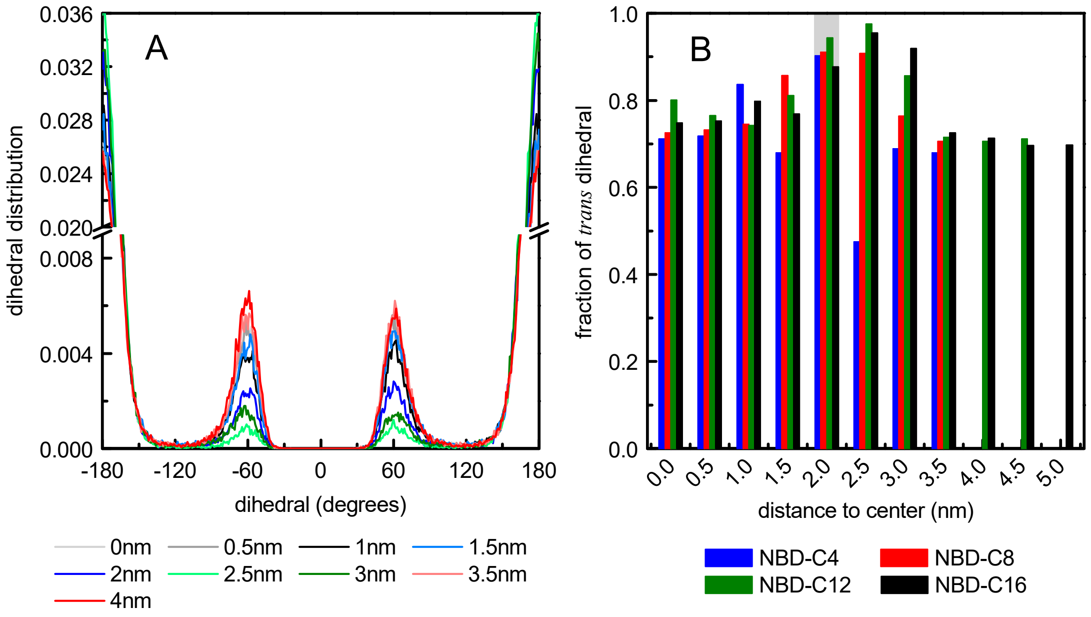


**Figure S10.** Chain dihedrals in SpM:Chol (6:4) bilayers. (A) distribution of the chain dihedrals of NBD-$C_{16}$, and (B) fraction of *trans* dihedrals of the alkyl chains of the NBD-$C_n$ amphiphiles, as function of the distance of the NBD to the bilayer center. Gray bar indicates the equilibrium position of the NBD group.

*4.3 Hydrogen Bonding*

An interesting parameter to characterize the interaction of this homologous series of amphiphiles with lipid membranes is the ability of the NBD group to form hydrogen bonds at different bilayer depths. The NBD fluorophore has an H atom bound to an amino N atom. This nitrogen can act as H-bond donor to water or to phospholipid atoms. On the other hand, there are several O and N atoms in the fluorophore, which can act as H-bond acceptors from water. For the following analysis, an H-bond for a given donor–H–acceptor triad was registered each time the donor–acceptor distance was < 0.35 nm and the H-donor-acceptor angle was < 30°. Figure shows the frequencies of H-bonding between NBD and either water or phospholipid (NBD NH as donor with POPC O, or SpM O/N atoms as acceptor in Figure (A, D, G, J), NBD NH as donor with $H_2O$ O as acceptor in Figure (B, E, H, K), and NBD N/O as acceptors with $H_2O$ OH as donor in Figure (C, F, I, L)). The profile of the H-bonding as a function of the position of the NBD group is similar for all amphiphiles, as expected from their similar structures. For long distances from the center of the bilayer, NBD is surrounded by water, and therefore does not establish hydrogen bonds with the phospholipids. At those positions, H-bonding to the water molecules is dominant (both considering NBD as donor or acceptor). As the NBD group approaches/inserts into the bilayer, the number of H-bonding of NBD NH with phospholipids' O atoms suddenly increases, becoming essentially permanent during the simulations. Near the equilibrium position of NBD (1 to 2 nm, with a broader region for pure POPC membranes), NBD NH makes preferentially hydrogen bonds with the phospholipids compared to water, in accordance to our previous simulations [3].

For the pure POPC membranes, at an intermediate position between the center of the bilayer and the equilibrium position, some degree of H-bonding between NBD NH and $H_2O$ O is observed, indicating the insertion of some water molecules in the membrane. Additionally, H-bonding between the N and O of NBD and water OH is significant for NBD positioned at all depths in the bilayer (except for $z$=0). When the NBD is in the center of the bilayer, the frequency of H-bonds, both to water and lipids, decreases significantly, indicating the lack of nearby groups capable of establishing H-bonds. In agreement with the lower deformation of the cholesterol-containing membranes, the probability of NBD to establish hydrogen bonds with the phospholipids, when

located close to the center of the bilayer, is reduced. Therefore, in opposition to that reported in MD simulations of phospholipid permeation [2], during the translocation process of the NBD-Cn molecules, a defect in the membrane may be created, but a pore is not formed.

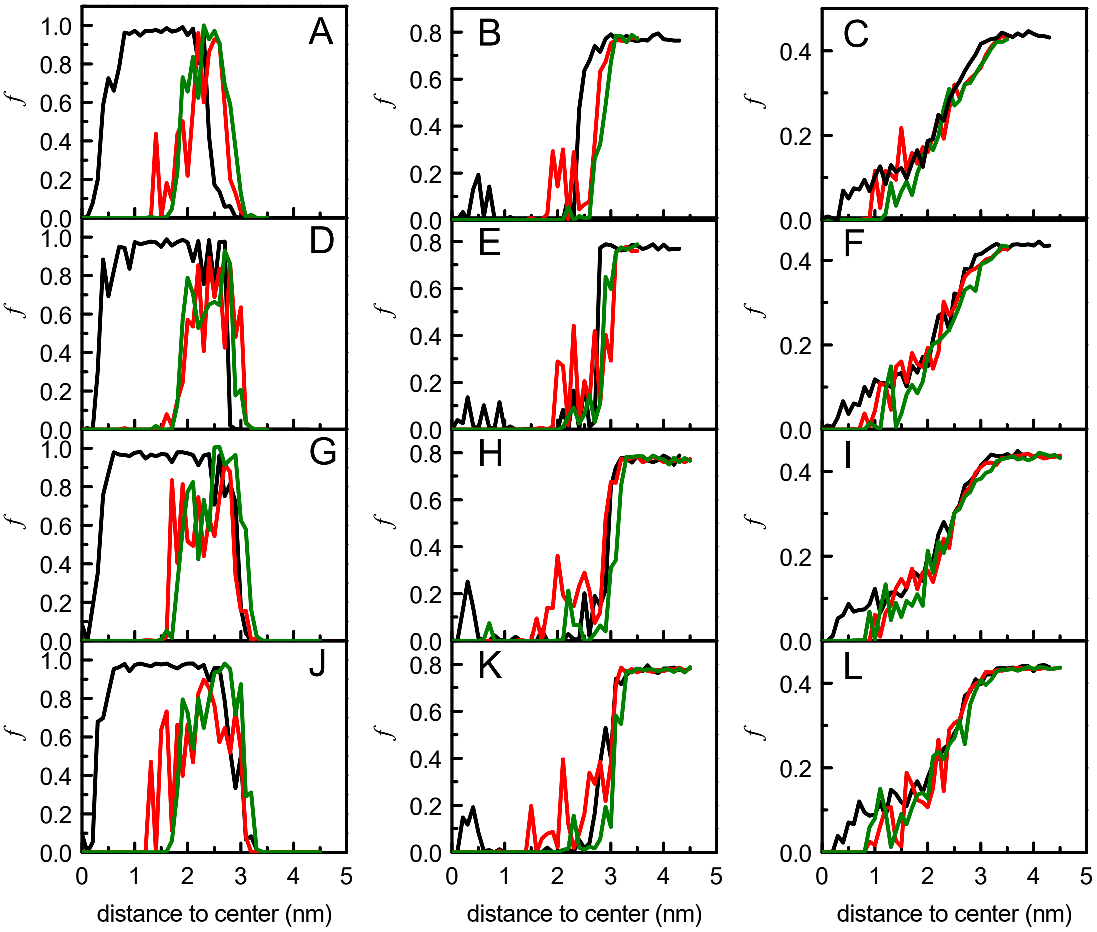


**Figure S11.** Average fraction of hydrogen bonds over the simulation time. For each amphiphile, NBD-C4 (A, B, C), NBD-C8 (D, E, F), NBD-C12 (G, H, I), NBD-C16 (J, K, L), data is shown for the simulation in POPC (black), POPC:Chol (1:1) (red) and SpM:Chol (6:4) (green). Hydrogen bonds are evaluated between the NBD and the lipid (A, D, G, J) and between the NBD and the water oxygens (B, E, H, K) or water hydrogens (C, F, I, L).

## *4.4 Orientation of the NBD Group*

The dipole moment of amphiphilic molecules like drugs may contribute importantly to their interaction with biomembranes. The NBD group has a dipole moment with an orientation very similar to the orientation of its short axis, defined as a vector between the C10 and the C11 atoms (pointing from the atom C10 to the atom C11, Figure S1). On the other hand, the lipid bilayer has

a strong dipole potential that interacts and influences the orientation of molecules/groups with large dipole moments. The dependence of the NBD orientation on the restrain position of the NBD in pure POPC bilayers is shown in Figure S12. In this analysis, an orientation of 180° means that the group $NO_2$ of the NBD is pointing to the water and an orientation of 0° means that the $NO_2$ is pointing to the center of the bilayer (or pointing to lower $z$ values). This orientation corresponds to a repulsive interaction between the dipole of the NBD-Cn and the lipid bilayer (parallel orientation) when the NBD group is at its equilibrium position in the POPC membrane, and is thus controlled by the orientation of the amphiphilic moment of the amphiphile and membrane [4]. The larger dipole potential of the cholesterol-enriched membranes increases the relative contribution of the interaction between the dipoles, and leads to the displacement of the NBD group towards a more external position in the membrane where the interaction between the dipoles becomes attractive.

When the NBD is restrained close to the center of the pure POPC bilayer (0.1. 0.5 nm) it tends, in all amphiphiles, to orient the $NO_2$ towards the center of the bilayer, thus presenting angle distributions around 40°. This is in agreement with the orientation of the negative pole of the amphiphile to the positive potential of the bilayer in the center of the bilayer. However, exactly in the center of the bilayers, the orientation of the NBD group is mostly random. As the restraint distance increases toward the water/membrane interface the hydrophobic chain serves as anchor and tends to orient the molecule according with the canonical orientation of the lipids in the bilayer, thus presenting angle distributions higher than 90°, with angles closer to 180° when the NBD is restrained near the desorption point. For higher distances, when the amphiphiles are in the water, the angle distributions are broad, covering all possibilities without an evident preference. The same behavior is observed at $z = 0$. This is common to all amphiphiles in all lipid bilayers. The well-defined orientation of NBD-C8 at $z = 0$ is an exception and should be interpreted as poor sampling.

The orientation of the NBD group when it is located close to the bilayer midplane in the POPC:Chol and SpM:Chol membranes should be considered with caution (see Figures S13 and S14). Although the dipole potential of the POPC:Chol membrane is higher than that of pure POPC, [4]the NBD does not show preferential orientation with the $NO_2$ group directed to the center of the bilayer. This may be explained by the higher compactness of the POPC:Chol membrane. In

order to orient the $NO_2$ toward the bilayer midplane, the alkyl chain has to visit the dense region of the bilayer in a more or less folded conformation. Therefore, the forces acting on the system will play into a compromise where the NBD is not allowed to correctly orient with the membrane dipole potential. This also seems to happen in SpM:Chol bilayers, for identical reasons.

At higher distances from the bilayer center, quantitative differences can also be found in the orientation of the NBD plane in POPC compared to POPC:Chol and SpM:Chol membranes. As the distance of the NBD to the bilayer center increases, it tends to orient with the $NO_2$ towards the water, as a consequence of the anchoring effect of the alkyl chain. However, in opposition to the pure POPC membrane where the transition from orientation angles ≤ 90° to angles close to 180° evolves smoothly, in the case of cholesterol-containing membranes there is a sharp variation. The need for the NBD to fit in the higher density of the cholesterol-containing bilayer (with the alkyl chain preferentially in the bilayer midplane) sharply leads to higher angles, closer to 180°.

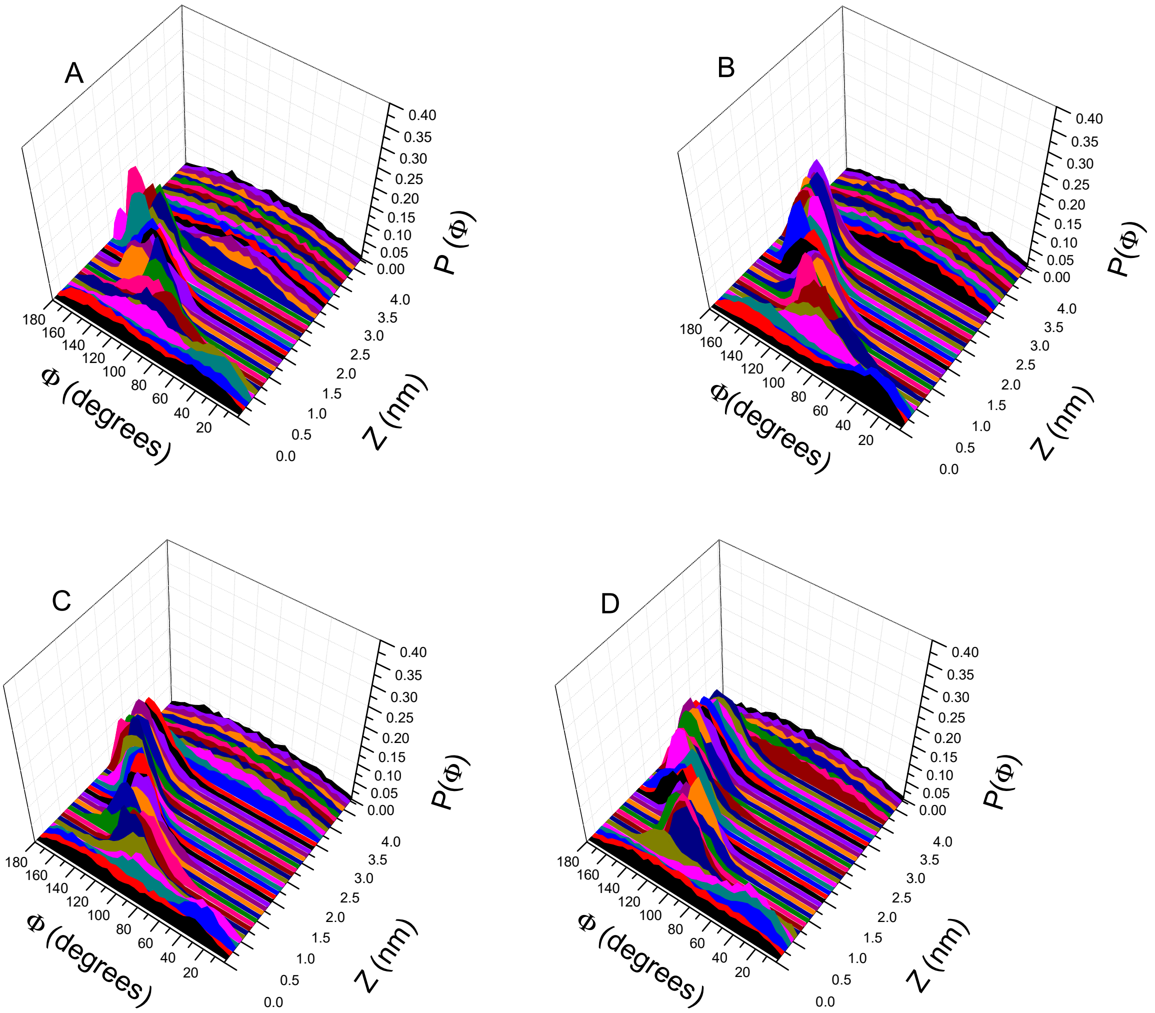


**Figure S12.** Orientation of the short axis of the NBD group (see definition in figure S1) at different bilayer depths in POPC bilayers for NBD-C4 (A), NBD-C8 (B), NBD-C12 (C) and NBD-C16 (D).

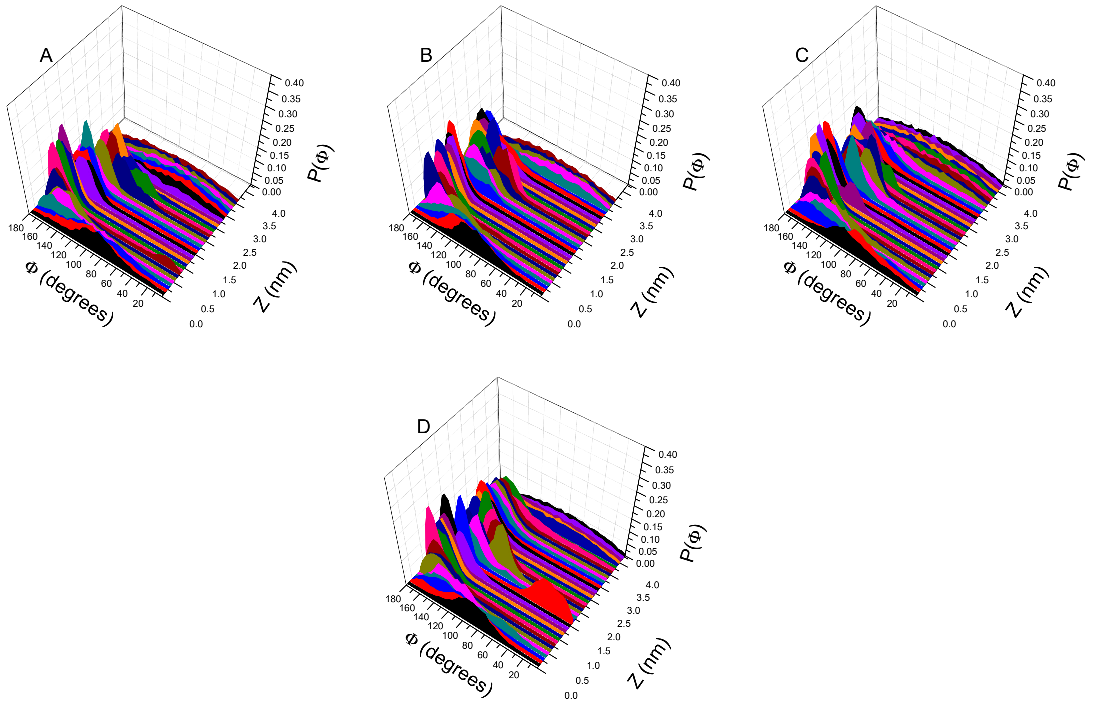


**Figure S13.** Orientation of the short axis of the NBD group (see definition in figure S1) at different bilayer depths in POPC:Chol(1:1) bilayers for NBD-C4 (A), NBD-C8 (B), NBD-C12 (C) and NBD-C16 (D).

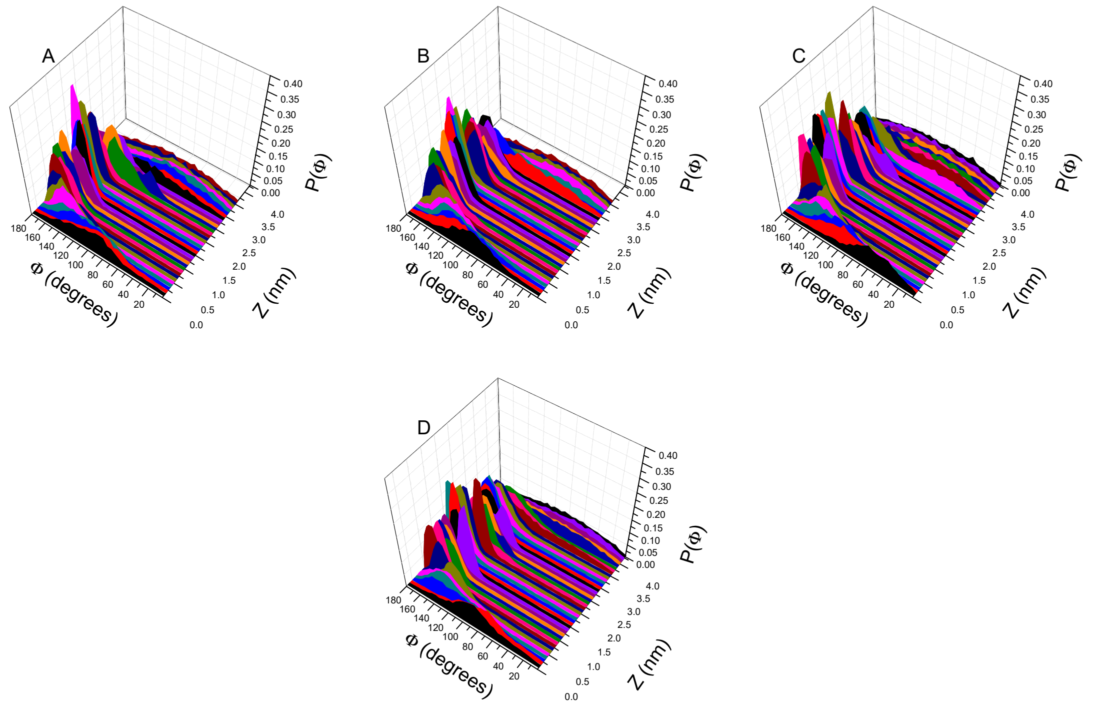


**Figure S14.** Orientation of the short axis of the NBD group (see definition in figure S1) at different bilayer depths in SpM:Chol(6:4) bilayers for NBD-C4 (A), NBD-C8 (B), NBD-C12 (C) and NBD-C16 (D).

## 5. Permeation of the Amphiphiles through the Lipid Bilayers

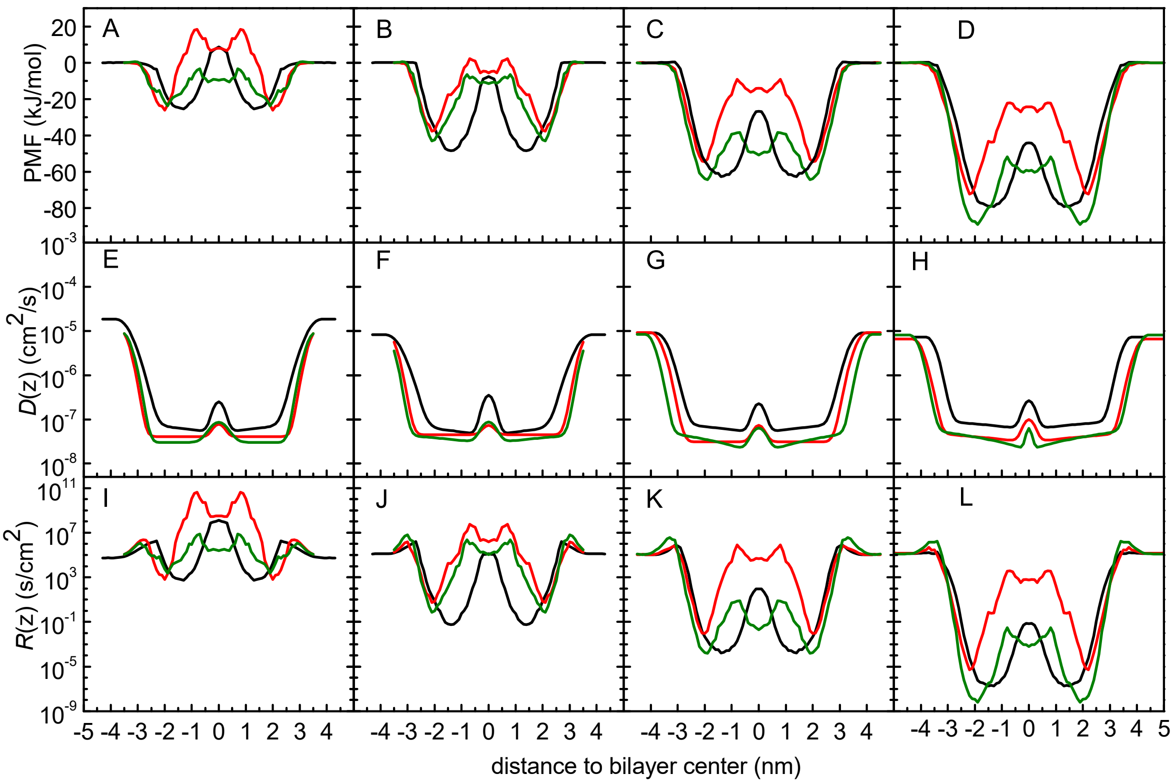


**Figure S15.** Free energy (a-d), local diffusion coefficient (e-h) and local resistance (i-l) profiles for the amphiphiles NBD-C4 (a, e, i), NBD-C8 (b, f, j), NBD-C12 (c, g, k) and NBD-C16 (d, h, l) in POPC (black), POPC:Chol (1:1) (red) and SpM:Chol (6:4) (green) bilayers. PMF profiles assume the energy reference on the water.

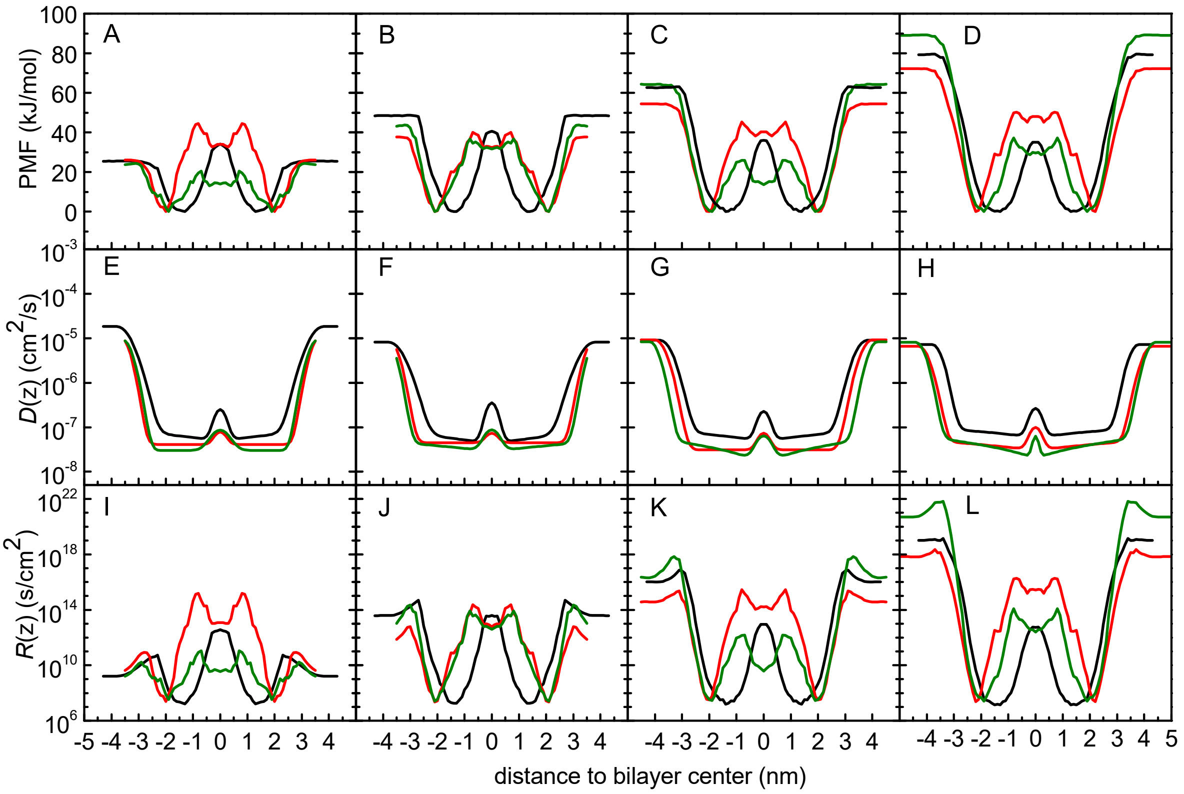


**Figure S16.** Free energy (a-d), local diffusion coefficient (e-h) and local resistance (i-l) profiles for the amphiphiles NBD-C4 (a, e, i), NBD-C8 (b, f, j), NBD-C12 (c, g, k) and NBD-C16 (d, h, l) in POPC (black), POPC:Chol (1:1) (red) and SpM:Chol (6:4) (green) bilayers. PMF profiles assume the energy reference on the equilibrium position of the amphiphiles.

## References


1. Filipe, H.A.L.; Moreno, M.J.; Róg, T.; Vattulainen, I.; Loura, L.M.S. How To Tackle the Issues in Free Energy Simulations of Long Amphiphiles Interacting with Lipid Membranes: Convergence and Local Membrane Deformations. *J. Phys. Chem. B* **2014**, *118*, 3572-3581, doi:10.1021/jp501622d.
2. Bennett, W.F.D.; MacCallum, J.L.; Tieleman, D.P. Thermodynamic Analysis of the Effect of Cholesterol on Dipalmitoylphosphatidylcholine Lipid Membranes. *J. Am. Chem. Soc.* **2009**, *131*, 1972-1978, doi:10.1021/ja808541r.
3. Filipe, H.A.L.; Moreno, M.J.; Loura, L.M.S. Interaction of 7-Nitrobenz-2-oxa-1,3-diazol-4-yl-Labeled Fatty Amines with 1-Palmitoyl, 2-Oleoyl-sn-glycero-3-phosphocholine Bilayers: A Molecular Dynamics Study. *J. Phys. Chem. B* **2011**, *115*, 10109-10119, doi:10.1021/jp203532c.
4. Cardoso, R.M.S.; Martins, P.A.T.; Ramos, C.V.; Cordeiro, M.M.; Leote, R.J.B.; Razi Naqvi, K.; Vaz, W.L.C.; Moreno, M.J. Effect of dipole moment on amphiphile solubility and partition into liquid ordered and liquid disordered phases in lipid bilayers. *Biochim. Biophys. Acta, Biomembr.* **2020**, *1862*, 183157, doi:https://doi.org/10.1016/j.bbamem.2019.183157.
